# Thioflavin-modified molecularly imprinted hydrogel for fluorescent-based non-enzymatic glucose detection in wound exudate

**DOI:** 10.1016/j.mtbio.2022.100258

**Published:** 2022-04-09

**Authors:** Giorgia Giovannini, Paolo Cinelli, Luciano F. Boesel, René M. Rossi

**Affiliations:** a Empa, Swiss Federal Laboratories for Materials Science and Technology, Laboratory for Biomimetic Membranes and Textiles, Lerchenfeldstrasse 5, CH-9014, St.Gallen, Switzerland; b Department of Trauma, University of Zurich, Zurich, Switzerland

**Keywords:** Fluorescence, Glucose detection, Molecularly imprinted hydrogel, Thioflavin, Boronic acid, Wound exudate

## Abstract

The concentration of glucose in the body's fluids is an important parameter that can indicate pathological conditions such as the progress of infected wounds. Several wearables and implantable detection approaches have been developed with high selectivity and sensitivity for glucose. However, all of them have drawbacks such as low stability, limited selectivity, and often require complex technology. In this work, we present a fluorescent-based cost-efficient imprinted hydrogel (MIH_GSH) capable of detecting glucose within 30 ​min. The imprinting approach allows us to improve the selectivity for glucose, overcoming the low specificity and limited binding efficiency at neutral pH of boronic acid-based detection mechanisms. The binding affinity determined for glucose-MIH_GSH was indeed 6-fold higher than the one determined for the non-imprinted hydrogel with a calculated imprinting factor of 1.7. The limit of detection of MIH_GSH for glucose in artificial wound exudate was calculated as 0.48 ​mM at pH 7.4 proving the suitability of the proposed approach to diagnose chronic wounds (*ca.* 1 ​mM). MIH_GSH was compared with a commercial colorimetric assay for the quantification of glucose in wound exudate specimens collected from hospitalized patients. The results obtained with the two methods were statistically similar confirming the robustness of our approach. Importantly, whereas with the colorimetric assay sample preparation was required to limit the interference of the sample background, the fluorescent signal of MIH_GSH was not affected even when used to measure glucose directly in bloody samples. The sensing mechanism here proposed can pave the way for the development of cost-efficient and wearable point-of-care tools capable of monitoring the glucose level in wound exudate enabling the quick assessment of chronic injuries.

## Highlights

• Highly sensitive and selective non-enzymatic approach to detect glucose in wound exudate.
• The fluorescent-based method ensured the detection of glucose in complex biological samples.
• The imprinting approach allowed overcoming the drawback of boronic acid-based methods.
• The cost-efficient approach is suitable for the development of point-of-care devices.

## Graphical abstract

**Figure:**
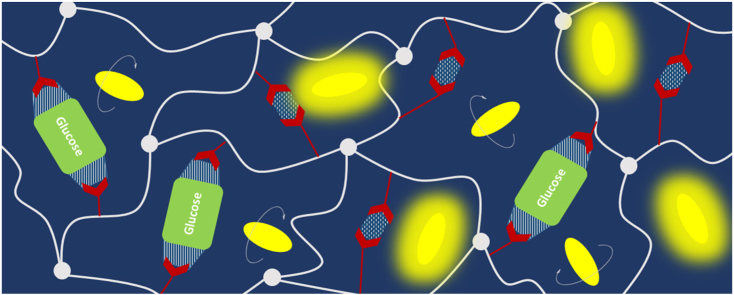

## 1 Introduction

Due to the inherent complexity of the wound healing process and the gap in evidence-based methods [1], wound management is based on non-optimized treatments that rely on trial-and-error approaches and unnecessary medical interventions. Therefore, the development of non-invasive technologies for monitoring wound healing seeks to facilitate prompt intervention favoring healing. In this context, the use of biomedical sensors for the proper management of wounds could have a crucial role in helping clinicians to monitor the healing process facilitating timely intervention in case of complications [2]. Many efforts have been invested to develop wound monitoring sensors capable of evaluating one or multiple physiological and chemical parameters such as pressure, pH, glucose, uric acid, and metalloproteinase [[3], [4], [5]]. Among these, glucose is not only an important biomarker of diabetic wounds [6] but it generally impairs wound healing when present at high concentrations, besides being used as a source of energy by bacteria to proliferate [7,8].

The relevance of glucose as a biomarker is reflected in the large literature reporting new or improved approaches to detect glucose with good sensitivity and selectivity. The mechanisms developed can be divided into two main groups: enzymatic and non-enzymatic methods. Enzymatic sensors consist of immobilized enzymes (e.g. glucose oxidase) on different substrates. The high specificity between enzyme and its substrate makes these approaches highly selective for glucose. However, the enzymatic activity is highly susceptible to environmental conditions hence their sensitivity is easily affected by variation of temperature, pH, and humidity [9]. The non-enzymatic approaches allow to overcome such drawbacks and are more stable and durable over time at the cost of reducing the selectivity for glucose [10]. Glucose non-enzymatic detection is foremost achieved using optical and electrochemical-based approaches. In optical sensors, the bunding between glucose and recognition moieties is translated into a measurable signal (e.g. fluorescence, absorbance, light scattering) [11]. This class of sensors has several advantages such as remote sensing, low cost, and fast response but the detected signal can be affected by environmental parameters (e.g. surrounding light, colored samples). Electrochemical sensors are based on the electrooxidation of glucose when interacting with electrodes [12]. The use of noble metals [13] and their composites [14] for the preparation of the electrodes allowed to improve the sensitivity to the electrooxidation of glucose. However, the blockage of the electroactivity by absorption on the electrode surface of undesired molecules (e.g., proteins) or solution active species (e.g., Cl^−^) affects the performance of such a technique [15].

Glucose-sensitive hydrogels (GSHs) have been largely studied for the design of enzyme-free sensors aiming to overcome the limits of the latter such as the low stability of the biological material. GSHs are formed by a crosslinked polymeric network containing glucose-binding moieties of boronic acid (BA) forming reversible hydrogen bonds with diols, and thus with glucose [16]. BA derivatives ensure reproducibility and stability of the detection system but with the disadvantage of the low selectivity for glucose towards other saccharides [17]. However, glucose forms reversible interactions with two moieties of BA when properly oriented and distributed in the hydrogel network [18]. Such double cross-linking has been exploited to improve the selectivity of the interaction for glucose among other carbohydrates. For instance, fructose, having only one set of *cis*-diol can only bind to one moiety of BA. BA molecules can also form reversible interactions with each other via hydrogen bonds [19]. The formation of such dimers is favored by specific conditions such as their concentration, orientation, pKa of the boronic acid, and the presence of donors in the structure suitable for additional intermolecular interactions [[20], [21], [22]].

Molecularly imprinting a polymeric network allows creating binding sites with high sensitivity and selectivity for the targeted molecule. In particular, the polymerization takes place in presence of the template (targeted analyte) thus after its extraction from the formed network, specific molecular recognition sites, complementary in shape, size, and functional groups to the imprinted molecule, are created. Molecular imprinting approaches have been employed for the recognition of glucose with good sensitivity and selectivity for the development of extraction mechanisms [23,24] or sensing platforms based on electrochemical signals [25,26] and Surface Plasmon Resonance (SPR) [27]. Thanks to their flexibility, swelling properties, and biocompatibility, hydrogels have been investigated as the substrate for molecularly imprinting approaches [[28], [29], [30]]. To detect glucose, BA moieties are often incorporated in the hydrogel network achieving glucose-sensitive molecularly imprinted hydrogels. As mentioned for GSHs, the re-adsorption of glucose in the specifically imprinted pockets leads to swelling/shrinkage of the hydrogel. This variation in volume has been transduced in different measurable signals. Even though the main mechanism of detection is based on the measurement of the refractive index (RI) variation with the changes in the hydrogel volume in response to glucose, other examples can be found in the literature. For instance, Wang et al. developed a glucose-imprinted hydrogel in which RI changes with the incorporation of glucose [49]. Xue et al. developed a glucose-responsive photonic crystal hydrogel in which diffraction red shifted in response to the glucose concentration [44] whereas Guo et al. recently developed plasmonic hydrogel-based optical fibers where the variation of the SPR enables glucose quantification [60]. Despite the good results achieved with such approaches, the variation of RI in response to the change of the hydrogel volume is small and the measurement requires sophisticated equipment. Moreover, the fabrication of colloidal crystal-based sensors is laborious and SPR analysis requires expensive instrumentation. In this view, fluorescent-based detection approaches are preferential since they are easily designed, simple to measure, cost-efficient, and have been therefore widely exploited for the development of detection systems [31].

Molecular rotors are a class of fluorophores with a rotation center in their structure that allows Twisted Intermolecular Charge Transfer (TICT). Since the rotation freedom is strongly related to the microenvironment of the molecule, a small change leads to a measurable variation of the fluorescent signal. In particular, at low viscosity, such fluorophores exhibit weak fluorescence since the excitation absorbed is released as kinetic energy due to the rotation freedom. On the contrary, with the increase of the viscosity, an increase in fluorescence intensity can be observed since the energy absorbed decays as a fluorescence rather than being dissipated as kinetic energy. The peculiar optical behavior of such molecular rotors have been widely used to analyze the viscosity of fluids and have found application in several research fields ranging from the quality control of silicone oils for mechanical engineering [32] to the characterization of the viscosity of cell's compartments and living tissues in biology [33]. Thioflavin T (Tht) is a fluorescent molecular rotor commonly used to image amyloids [34]. While binding to amyloids, molecular rotation is inhibited and Tht establishes a strong red-shift of its excitation/emission spectra with the appearance of a characteristic fluorescent peak at 480 ​nm [35].

**Fig. 1:**
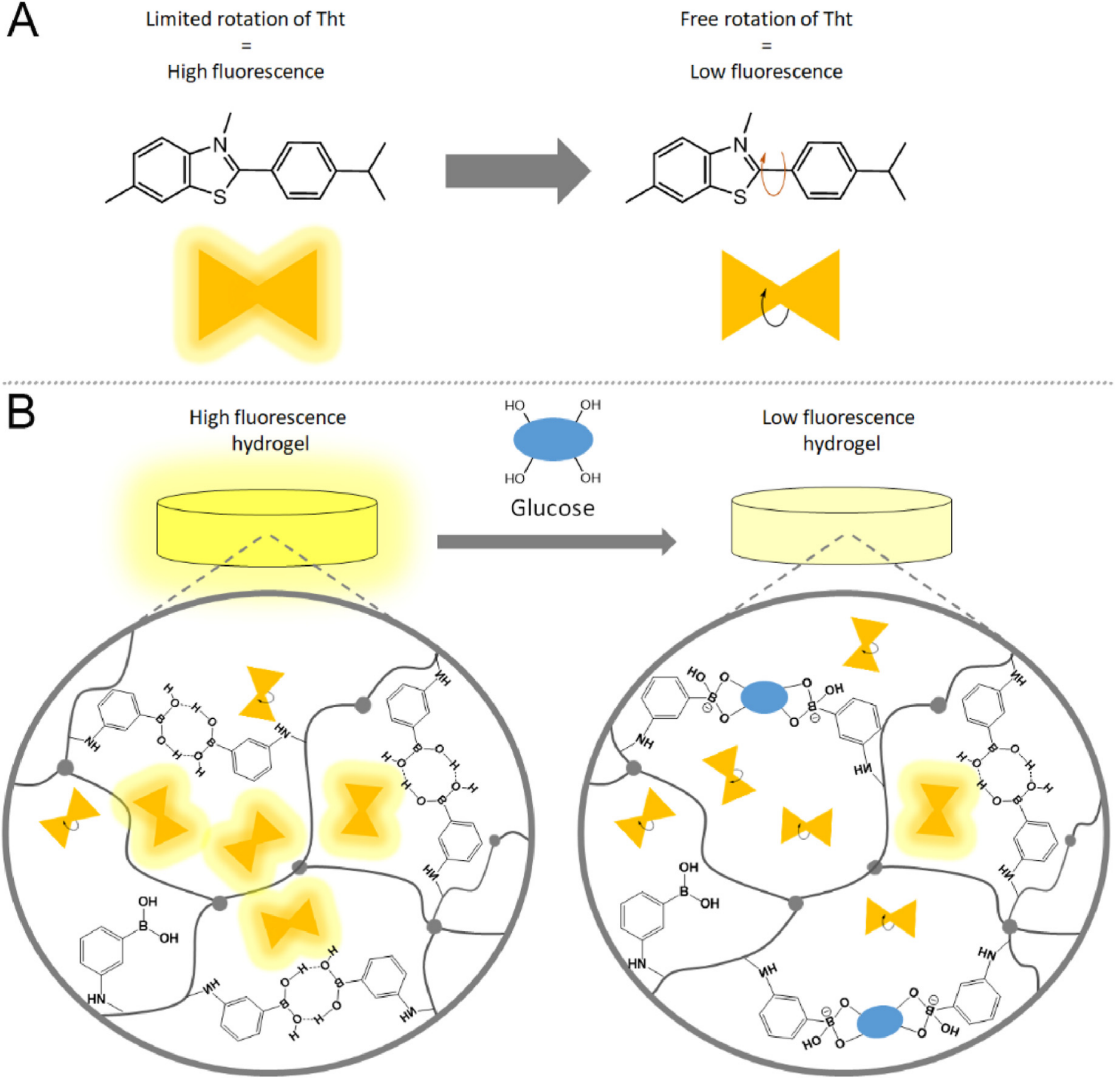
Scheme of the detection mechanism of MIH_GSH. A) Chemical structure of Tht and rotation center which leads to the change in fluorescence. B) Mechanism of the fluorescent-based glucose detection of the MIH_GSH.

In this work, we fabricated a non-enzymatic fluorescent-based imprinted GSH glucose sensor. The designed platform combines the stability of non-enzymatic GSH with the improved selectivity towards glucose achieved using molecularly imprinted hydrogel (MIH). Furthermore, the incorporation of Tht allows transducing the glucose concentration into a change of the fluorescent signal ensuring an easy and cost-efficient quantification of glucose. The MIH_GSH is an acrylamide-based hydrogel formulated using 3-acrylamide-phenyl boronic acid (AAPBA) that increases the hydrogel stiffness due to the formation of dimers through intramolecular H-bonds formation. The scheme of the detection approach is reported in Fig. 1. Glucose (template) was added to the mixture of acrylates during the polymerization of the hydrogel and it was then removed leaving pockets optimized to accommodate glucose in the matrix. Once glucose is re-absorbed in the hydrogel network, it interacts with the boronic acid moieties displacing intramolecular H-bonds. The loosening of the internal interactions in the hydrogel network leads to a decrease of the hydrogel stiffness which is transduced into a decrease of the Tht fluorescent peak at 480 ​nm. The selectivity for glucose was improved by exploiting the combinational effect of the multivalency of BA-glucose and the imprinting approach that ensure the appropriate orientation of the BA units in the pockets, favoring the 2:1 interaction in contrast to the monovalent interaction (1:1) observed instead with fructose and other carbohydrates.

## 2 Materials and methods

### 2.1 Materials

Acrylamide (AA), bisacrylamide (Bis), ((2-Dimethylaminoethyl) methacrylate (DMAEMA), and tetramethylethylendiammine (TEMED), HEPES sodium salt, DMEM D5030, Sodium bicarbonate (NaHCO_3_), d-glucose, lactate, colorimetric glucose assay kit (GAGO20), NaCl, KCl, Na_2_HPO_4_, KH_2_PO_4_, NaOH and HCl were purchased from Sigma-Aldrich. Thioflavin T (Tht) and 3-acrylamide-phenyl boronic acid (AAPBA) were purchased from TCI. Porcine serum was purchased from Thermo Fisher Scientific. Ammonium persulfate (APS), d-mannose, d-lactose, and d-fructose were purchased from Fluka.

### 2.2 Methods

#### 2.2.1 Synthesis of the imprinted GSH

Stock solutions in HEPES were prepared as follows: AA 30%, Bis 2%, Tht 2.5 ​mM, TEMED 6.67 ​mM, and APS 100 ​mM. AAPBA was solubilized in DMSO at a concentration of 1.6 ​M (sonication required to achieve a clear yellowish solution). Glu was solubilized in HEPES at the concentration of 0.8 ​M. The AAPBA and Glu solutions were mixed reaching a final concentration of 0.8 and 0.4 ​M respectively for AAPBA and Glu. AAPBA-Glu was mixed in dark and at room temperature for 2 ​h and then used for the formulation of MIH_GSH. For the preparation of NIH_GSH instead, AAPBA was diluted with HEPES to 0.8 ​M and mixed for 2 ​h. Defined volumes of the so prepared stock solutions were vortexed together, achieving the final concentrations mentioned in Table 1. For instance, AA 30% 0.38 ​mL, Bis 2% 0.31 ​mL, Tht 0.2 ​mL, TEMED 20 ​μL, APS 50 ​μL, DMAEMA 54.8 ​μL and 0.4 ​mL of AAPBA (NIH_GSH) or AAPBA-Glu (MIH_GSH) adding HEPES up to 2 ​mL as final volume. After mixing, the samples were poured into Petri dishes. Polymerization occurred overnight under vacuum. The polymerized hydrogel was washed with DI water and then incubated with DI water (10 ​mL) for 2 ​h (x 2) and with PBS pH4 (10 ​mL) for 4 ​h (x2), then being left in DI water overnight. Tht not incorporated into the hydrogel matrix was removed with the first washing step. The hydrogel was then cut into 8 identical pieces which were placed in a 96-well plate. The hydrogel was equilibrated and kept hydrated until the experiment.

**Table 1:** Glucose quantification in WE samples using the proposed fluorescent-based method compared with the results achieved with a commercial colorimetric assay. Values are reported as mean ​± ​sd (MIH_GSH n ​= ​6; colorimetric assay n ​= ​3). Statistic analysis indicated that the single measures are not statistically different from the means (two-samples *t*-test, significance level 0.05, confidence level 95%, actual power).

Glucose [mM]	S-1	S-2	S-3	S-4	S-5
**MIH_GSH**	8.8 ​± ​1.9	4.2 ​± ​0.8	2.1 ​± ​0.7	4.0 ​± ​1.0	0.4 ​± ​0.8
**Colorimetric assay**	8.9 ​± ​0.4	5.4 ​± ​0.2	1.8 ​± ​0.6	5.0 ​± ​0.6	0.3 ​± ​0.0

#### 2.2.2 Buffers used

*HEPES* 1 ​mM pH8*:* HEPES sodium salt (0.26 ​g) was dissolved in 1L of deionized water and the pH was adjusted to 8 using NaOH 1 ​M.

*Phosphate Buffered Saline* 10 ​mM *(PBS):* NaCl 0.137 ​M, KCl 0.0027 ​M, Na_2_HPO_4_ 0.01 ​M, KH_2_PO_4_ 0.0018 ​M solubilized in deionized water. The desired pH was reached by adding NaOH 1 ​M or HCl 1 ​M.

*Artificial Wound exudate (AWE):* DMEM D5030 8.3 ​g/L, NaHCO_3_ 3.7 ​g/L, and porcine serum 10% v/v were solubilized in MilliQ water up to 1L. The desired pH was reached by adding NaOH 1 ​M or HCl 1 ​M.

*Wound exudate (WE):* Wound exudates were collected from negative-pressure wound therapy sponges upon exchange in the operation room. The wound exudate was collected by squeezing vacuum-assisted closure (VAC) sponges previously applied on unhealed wounds. The collection was approved by the Cantonal Ethics Commission, Zurich (BASEC Nr. 2021-00910).

#### 2.2.3 Leakage experiment

Once synthesized, MIH?GSH and NIH_GSH were firstly rinsed once with water to remove Tht that was not incorporated inside the matrix, as part of the synthetic procedure. Subsequently, both hydrogels were soaked in water (3 ​mL). After 1, 2, 3, 24, and 48 ​h the water was collected and replaced with a fresh liquid (3 ​mL). The amount of Tht that leaked out of the hydrogel was quantified by measuring the absorption signal of Tht in the water collected at each washing step. The amount of Tht (nmol) was calculated using the calibration curve of Tht in water (slope 0.0073; intercept 0.0042; R^2^ 0.99). Given that 500 ​nmol (250 ​μM in 2 ​mL) were added for the hydrogel formation, we calculated the % of Tht that leaked out of the hydrogels during washing.

#### 2.2.4 Scanning electron microscopy (SEM) analysis

SEM images were acquired using a Hitachi S-4800 scanning electron microscope at an acceleration voltage of 5 ​kV and magnifications from 600 to 40 k. The samples were lyophilized and then fixed on conductive carbon tape. To facilitate imaging, samples were sputter-coated with 7 ​nm of Au/Pd alloy.

#### 2.2.5 Nitrogen sorption measurements

Nitrogen sorption measurements were used to determine the surface area and pore volume of the hydrogels (MIH_GSH and NIH_GSH), which were previously freeze-dried to remove all water content. The samples were further dried at 80 ​°C for 20 ​h before the introduction of N_2_ gas. The nitrogen adsorption/desorption cycles were measured at 77 ​K using a Micromeritics Triflex. Brunauer-Emmet-Teller (BET) method was used to calculate the specific surface area. The pore size, volume, and surface area of pores were obtained from analysis of the adsorption branch using the Barrett-Joyner-Halenda (BJH) method.

#### 2.2.6 FT-IR analysis

IR spectra were recorded with a Varian 640-IR, software Aligent Resolutions PRO. Specimens of MIH_GSH and NIH_GSH before and after washing were lyophilized before the analysis.

#### 2.2.7 Swelling study

To determine the swelling of the hydrogels at different pHs, each specimen was soaked in the solvent and weighed at specific intervals (0, 0.5, 1, 2, 4, and 12 ​h). The %swelling was calculated considering the variation of the weight at each time point (W_n_) to the initial weight (W_i_) using the following equation:

$$\%swelling=\left(\frac{Wn-Wi}{Wi}\right)100$$

#### 2.2.8 UV–Vis analysis

UV–Vis spectra of Tht and Tht-containing hydrogel were recorded using Varian Cary 50Bio connected to 50 MPR (Agilent) in a 96-well plate.

The quantification of glucose with colorimetric assay (Sigma-Aldrich): 60 ​μL of WE samples (dilution factor 10) or glucose solution in PBS were added to a 96-well plate. 120 ​μL of the reagent (containing glucose oxidase, peroxidase, and o-dianisidine) was added to each well and the plate was incubated at 37 ​°C. After 30 ​min the reaction was stopped by adding 120 ​μL of 6 ​M ​H_2_SO_4_ and the absorbance was measured at 540 ​nm. To quantify the amount of glucose in WE samples a calibration curve was used.

#### 2.2.9 Rebinding experiment

To determine the amount of glucose absorbed by MIH_GSH and NIH_GSH, the signal recorded for the initial concentration (*Ci* ​= ​2.5 ​mM) was used as reference (*Ab*_*ref*_). The amount of glucose that was not absorbed by the hydrogel (*Cf*) was calculated with the following formula:

$$Cf=\left(\frac{Ab_{sample\;Ci}}{Ab_{ref}}\right)$$

The imprinting factor was calculated as the ratio between *Q*_*MIH_GSH*_ and *Q*_*NIH_GSH*._ Q was calculated using equation (1) where *Ci* is the initial concentration of glucose used and Cf is the amount of glucose left in the supernatant, *M* is the mass of the specimen of the hydrogel and *V* is the volume of the glucose solution used during the experiment (200 ​μL).

Determination of the association binding constant (Ka):

Apparent binding affinities of the hydrogels were determined by measuring the variation of the fluorescent signal of the hydrogels when treated with different concentrations of different carbohydrates. Specimens of MIH_GSH and NIH_GSH were treated with 100 ​μL of carbohydrate solutions (i.e. d-glucose, d-mannose, d-lactose, and d-fructose) at different concentrations (0, 0.6, 1.25, 2.5, 5, and 10 ​mM; PBS pH 7.4). After 30 ​min of equilibration the fluorescent signal of the hydrogel was measured (λex 410 ​nm; λem 480 ​nm). Lineweaver-Burk plots were used to determine the Ka of MIH_GSH and NIH_GSH for each carbohydrate as the ratio between the increment and the slope of the curves. Lineweaver-Burk curves were obtained by plotting the invert of the signal variation (1/Δ*I*) on the y-axis and the invert of the carbohydrate's concentration (1/Q) on the x-axis.

#### 2.2.10 Fluorescent analysis

Fluorescent spectra were recorded adding samples of Tht or specimens of hydrogel (MIH_GSH or NIH_GSH) in a 96-well plate. 100 ​μL of solution at different concentrations of glucose (PBS, AWE, wound exudate) were added to each well. After 30 ​min of equilibration, the spectra of the samples excited at 410 ​nm were recorded and the intensity at 480 ​nm was used as the outcome value for data analysis. The relative fluorescence was used to compare the results achieved with different specimens and was calculated as the ratio between the initial value (*Ii*) and the intensity measured after 30 ​min of treatment with glucose solution (*In)*. Calibration curves were obtained using the -log(*I*_480_). All spectra were recorded using Varian Cary Eclipse (Agilent).

#### 2.2.11 Statistic analysis

Statistical analysis was accomplished using Origin 2020b software. To compare different samples one-way ANOVA (significance level 0.01) was used or, when specified, one-way ANOVA with a post hoc test to define the relationship between groups (significance level 0.05, Tukey test, Levene's test, actual power). To evaluate the difference between the means of two samples we used two-sample *t*-test (null hypothesis: mean1 – mean2 ​= ​0; alternative hypothesis: mean1 – mean2 <> 0; significance level 0.05, confidence level 95%; actual power analysis) to define the discrepancy between sets of data.

## 3 Results and discussion

### 3.1 Formulation of the hydrogel

The formulation of MIH_GSH was defined to obtain a final material with specific morphology. Indeed depending on the porosity, glucose (Glu) can diffuse more or less easily through the thick matrix and interact with PBA moieties [36]. The porosity of the hydrogel network is mainly determined by the rate of cross-linking that can be defined by changing parameters such as the concentration of monomers, crosslinker, initiator, and catalysts. In this work, the network is formed by using acrylamide (AA) and bisacrylamide (Bis) with the addition of the PBA as the glucose-sensing moiety and (2-Dimethylaminoethyl) methacrylate (DMAEMA) as a cationic derivative which reduces the pH sensitivity of PBA-based glucose detection approaches [37]. Free radical polymerization is induced by using ammonium persulfate (APS) and tetramethylethylendiamine (TEMED) as initiator and catalyst respectively in the reaction. The concentration of all components was defined to have a hydrogel with small pores size allowing the diffusion of glucose but, at the same time, the formulation was defined to have a mechanically stable hydrogel. In this way, we achieved a system in which the presence of glucose leads to variations of the hydrogel internal structure at the molecular level. Aiming to maximize Tht rotation in response to nearly imperceptible environmental variations, we settled on simply entrapping physically free Tht into the rigid matrix of the hydrogel. Thanks to this expedient, we were able to convert minimal changes of stiffness into a measurable signal given the glitches in the fluorescence variation of Tht observed when it is conjugated [38] or chemically modified [39]. With a mechanically rigid matrix, we limited the swelling and the variation of the internal structure of the hydrogel which could lead to an undesired oscillation of Tht's fluorescence regardless of glucose. Moreover, the stiffness of the hydrogel matrix ensured the stable incorporation of Tht as confirmed by the leakage study (Table S 2)." To maximize the imprinting efficiency, AAPBA was pretreated with glucose (ratio 2:1) in HEPES 1 ​mM at pH 8. After 2 ​h of agitation, the mixture was added to the hydrogel formulation reaching a final concentration of 160 ​mM of AAPBA and 80 ​mM of the carbohydrate. For the preparation of the non-imprinted hydrogel (NIH_GHS), AAPBA was pretreated without glucose addition. Lastly, TEMED and APS were added to the mixture. The so achieved solution was then quickly vortexed, poured into molds, and left to polymerize under vacuum overnight. The chosen hydrogel formulation is reported in Table S 1. The excess reactants and Tht were removed by washing and repeated incubations in DI water. The hydrogel was then incubated for 4 ​h at room temperature in PBS pH4 to remove the template since the interaction between AAPBA and glucose is unfavored in an acid environment. The hydrogel was then cut into 8 identical specimens and placed in a 96-well plate in which all experiments were accomplished using a plate reader.

### 3.2 Hydrogel characterization

**Fig. 2:**
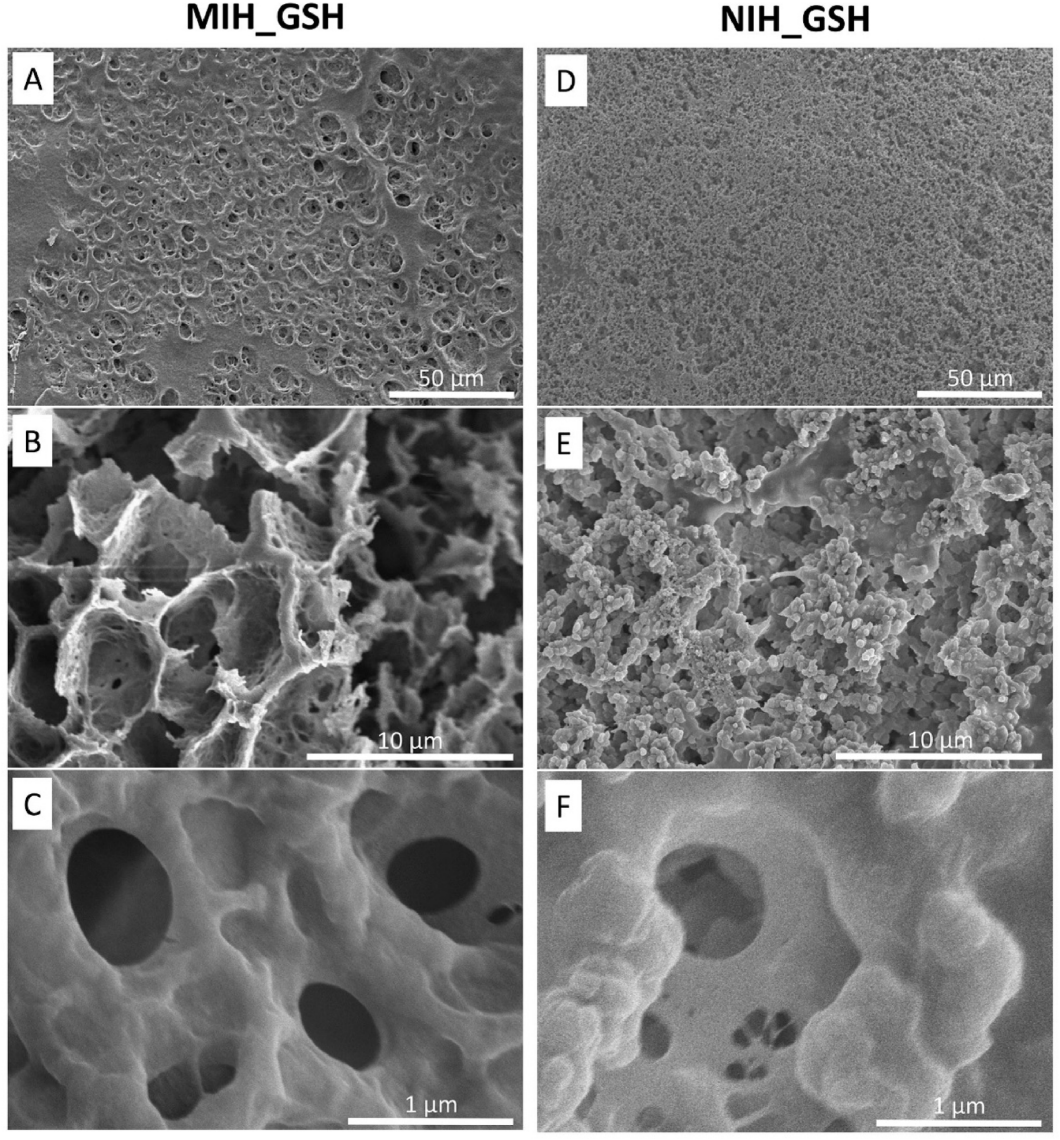
SEM analysis of pores and morphological structure of MIH_GSH and NIH_GSH hydrogels. Images were acquired using different magnitudes: ×600 (A and D), x5k (B and E), x40k (C and F).

The hydrogel was characterized morphologically by scanning electron microscopy (SEM). Fig. 2 shows the micrographs of the MIH_GSH and NIH_GSH after washing. The imprinting leads to a variation of the porosity of the polymeric network and the arrangement of the material. MIH_GSH showed a more porous network compared to NIH_GSH (Fig. 2 A and D), which is likely related to the presence of glucose during polymerization. The apparent structural difference noticeable between the polymeric network of MIH_GSH (linear) and NIH_GSH (globular) can be explained by the different orientation of the AAPBA when free (NIH) or linked to glucose (MIH) during polymerization of the acrylic derivatives (Fig. 2 B and E). At the micrometer level, both hydrogels showed pores with a diameter of ca. 1 ​μm ​at the surface (Fig. 2C and F). On the other hand, analyzing both imprinted and non-imprinted hydrogels by N_2_ sorption method, we proved that this formulation allows achieving a matrix characterized by a high density of pores at the nanometer range. In the case of NIH_GSH, the pores were of such a small size that N_2_ sorption analysis was non-trivial (limit of detection 2–200 ​nm). Such a tight network limits the diffusion of large molecules (e.g. proteins) present in the wound exudate avoiding undesired alteration of the detection mechanism but it ensures the diffusion of small molecules such as glucose (diameter *ca.* 1 ​nm) [40].

Imprinted and non-imprinted hydrogels were further characterized by Fourier transform infrared spectroscopy (FT-IR). The strong and very broad-ended band visible near 3600-3400 ​cm^−1^ in both NIH_GSH and MIH_GSH (before and after washing) represents the OH stretching band of AAPBA and indicates the presence of intermolecular hydrogen-bonding [41,42] (Figure S 1-A). The effective removal of glucose after washing is confirmed by the disappearance of peaks indicating C–B stretching (1016 ​cm^−1^) and B–O–H deformation (955 ​cm^−1^) after washing MIH_GSH, and their absence in NIH_GSH (Figure S 1-B) [43].

**Fig. 3:**
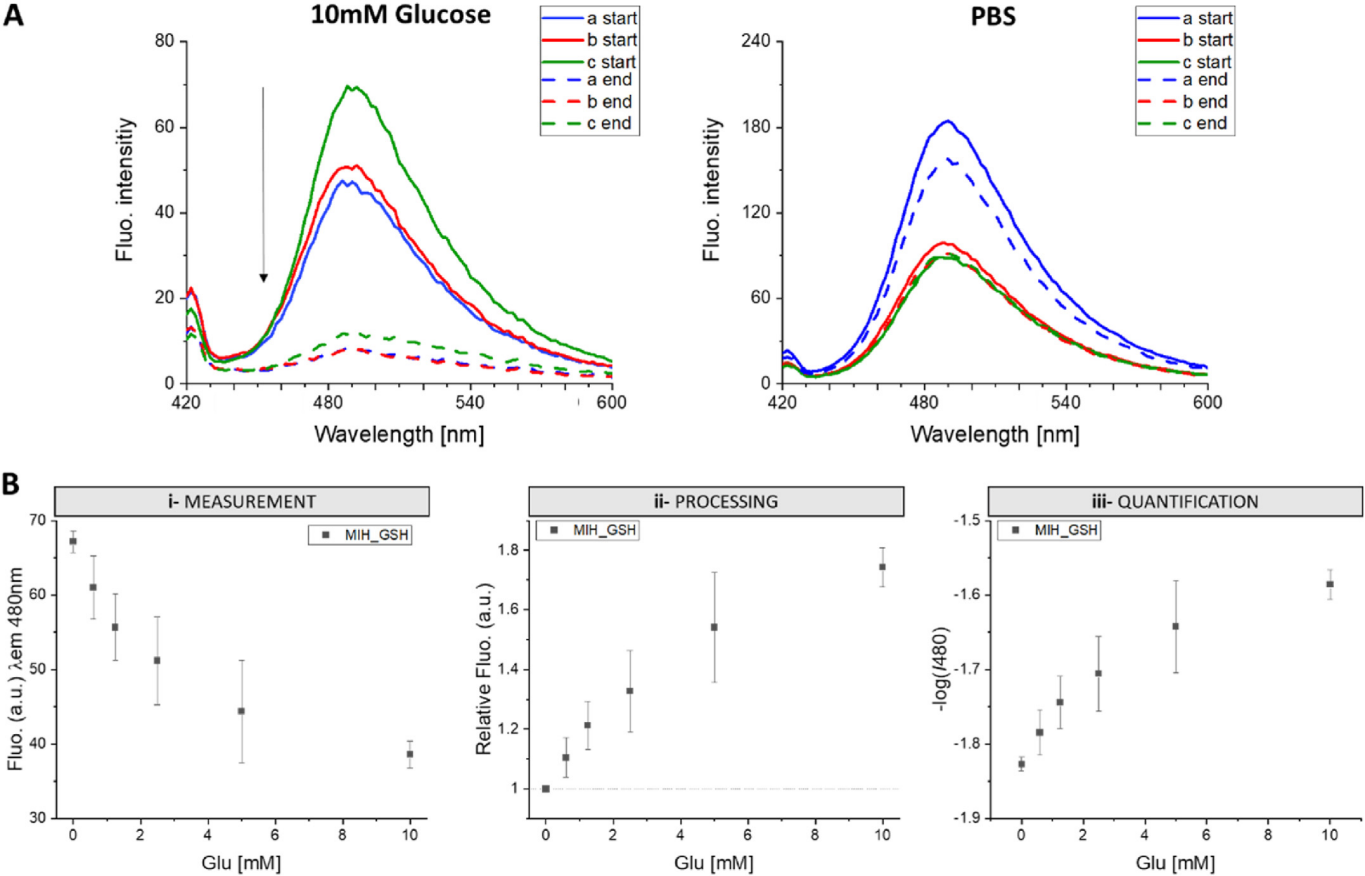
*A) Confirmation of the detection mechanism with three independent specimens of MIH_GSH (indicated as a, b and c). The fluorescence intensity of the hydrogel measured at* 480 ​nm *decreases only when treated with glucose (right) otherwise it is stable except for sample a for which a small decrease of the signal was observed but it was not comparable with the decreases induced by glucose (left). B) Evaluation process: measurement of the fluorescent signal (excitation* 410 ​nm*, emission* 480 ​nm*) in response to glucose concentration (i-measurement); Calculation of the relative fluorescence to better compare the results achieved within different specimens (relative fluorescence ​=* ​Ib_480_/In_480_*) (ii-processing); Quantification of the signal using the -log of the intensity measured (-logI480) in order to achieve a positive slope allowing the calculation of the limit of detection of the approach and to quantify Glu in unknown samples (iii-quantification). Values are expressed as mean ​± ​sd (n ​= ​6).*

An important characteristic of the GSHs here synthesized is related to their optical properties which determine the sensing mechanism. Due to the low transparency of the hydrogel, the UV–Vis measurement was carried on a thin slice of the hydrogel allowing to observe that the maximum absorbance peak of Tht 410 ​nm in the hydrogel network becomes broader compared to the signal of Tht in solution (Figure S 2-A), whereas the typical Tht fluorescent peak was only measured when the fluorophore was embedded into the hydrogel, as expected (Figure S 2-B). The effect of the formation of H-bonds between AAPB moieties on the hydrogel stiffness was confirmed by the increase of the fluorescent signal measured for MIH_GSH after glucose removal (Figure S 2-C), whereas the signal of NIH_GSH slightly decreased due to the settlement of the hydrogel network. To confirm the glucose responsiveness, the hydrogels were treated with a solution of glucose (10 ​mM) and with PBS. As hypothesized, the fluorescent signal at 480 ​nm significantly decreases only in presence of glucose, as reported in Fig. 3-A.

The formulation of the hydrogel was defined to limit the pH dependency that commonly affects boronic acid-based detection methods, ultimately improving the robustness of glucose detection. As reported in the literature, the effect of the pH in the detection efficacy is a common limitation of non-enzymatic approaches [44]. The use of the cationic acrylate derivative DMAEMA reduces the pKa of the boronic acid allowing the interaction with glucose to occur at physiological pH range (pH 6–7) rather than at pH ​> ​8 [45,46]. Furthermore, the presence of DMAEMA allows controlling the pH-dependent swelling of acrylic hydrogel [47] which otherwise would interfere with glucose quantification. The swelling behavior of MIH_GSH and NIH_GSH was studied and the results proved that at all pHs tested (5, 6, and 7) both hydrogels shrink similarly, with shrinkage of about 30–40% for MIH_GSH and about 40–50% for NIH_GSH after 4 ​h (Figures S 3-A and B). Between 4 and 12 ​h, the samples did not shrink much further. It is worth mentioning that the swelling study was conducted on already hydrated hydrogels, which were then treated with PBS at different pHs. Therefore, the shrinking of the hydrogel could have been eventually related to the change in pH and not to the rehydration. According to the results of the swelling study, before each experiment, the hydrogels were equilibrated with PBS at pH 7.4 for 24 ​h to stabilize the network. Monitoring the changes of the fluorescent signal at 480 ​nm of imprinted hydrogels once treated with different concentrations of glucose, we observed that a plateau is achieved after a few minutes (Figures S 4-A). However, the reversible interaction between glucose-boronic acid leads to fluctuation of the signal. Statistical analysis of the signal measured at different time points (15, 30, 45, and 60 ​min) indicated that the signal did not change significantly therefore we selected 30 ​min as the incubation time for each experiment (Figure S 4-B). The chosen detection time allowed to achieve a linear variation of signal in response to the glucose concentration (Figure S 4-C) and it is comparable to similar methods reported in the literature [48,49].

The influence of the environmental pH on the sensitivity of detection was further evaluated by treating MIH_GSH and NIH_GSH with a 5 ​mM solution of glucose in water, PBS, and artificial wound exudate (AWE) [50] adjusting to pH 6.2 and 7.4 which are typical conditions present in wounds [51]. Statistical analysis between the values measured at the two different pH, for water and PBS, indicated a good reproducibility without a significant difference from the mean values (p ​= ​0.05; Figures S 5-A and B). On the contrary, a statistical difference was observed between the values measured in AWE at pH 6.2 and 7.4 for both MIH_GSH and NIH_GSH (Figure S 5-C). This difference can be explained by the effect of the pH on the components of the AWE. For instance, proteins might be subject to denaturation/aggregation at neutral conditions altering the absorption of glucose by the hydrogel. The stability of the hydrogels was confirmed by measuring the signal at the beginning and the end of an experiment after the removal of the glucose bonded during the experiment. The fluorescent signals measured before and after the experiment were not statistically different, confirming the stability of the detecting platform over time and the possibility of reusing the same specimens for several tests (p ​< ​0.05, Figure S 6).

### 3.3 Glucose-sensing evaluation

The responsiveness of the here synthesized hydrogels was evaluated using glucose solutions in the concentration range of 0–10 ​mM, taking into account the values reported in the literature on glucose concentration in human wounds and considering that the presence of blood mixed with the exudate can lead to a higher concentration compared to the expectation [52]. The same protocol has been followed for all experiments: specimens of MIH_GSH and NIH_GSH were firstly hydrated overnight in PBS and then treated with 100 ​μL of the tested solution at the desired glucose concentration to completely cover the surface of the hydrogel in the well plate used. After 30 ​min of equilibration, the signal was measured (excitation 410 ​nm-emission 480 ​nm). After the measurement, the specimens were washed for 10 ​min with the same solvent as used during the experiment (water, PBS, or AWE at the specific pH) before testing the next sample (Fig. 3-B i). The initial fluorescence can vary slightly between samples and specimens due to some variations in the stiffness of the hydrogel matrix (H-bonds formation between boronic acid moieties). Therefore, the results were evaluated considering the relative fluorescent signal calculated as the ratio between the fluorescent signal measured for the blank (*Ib*_*480*_*,* 0 ​mM of glucose) and the one measured with glucose solutions (*In*_*480*_) (Fig. 3-B ii). Moreover, due to the saturation of the fluorescent signals with the increase in glucose concentration, the quantification of glucose was achieved considering the -log of the fluorescent values measured at 480 ​nm leading to linear regression curves with positive slopes suitable for further calculations (Fig. 3-B iii).

**Fig. 4:**
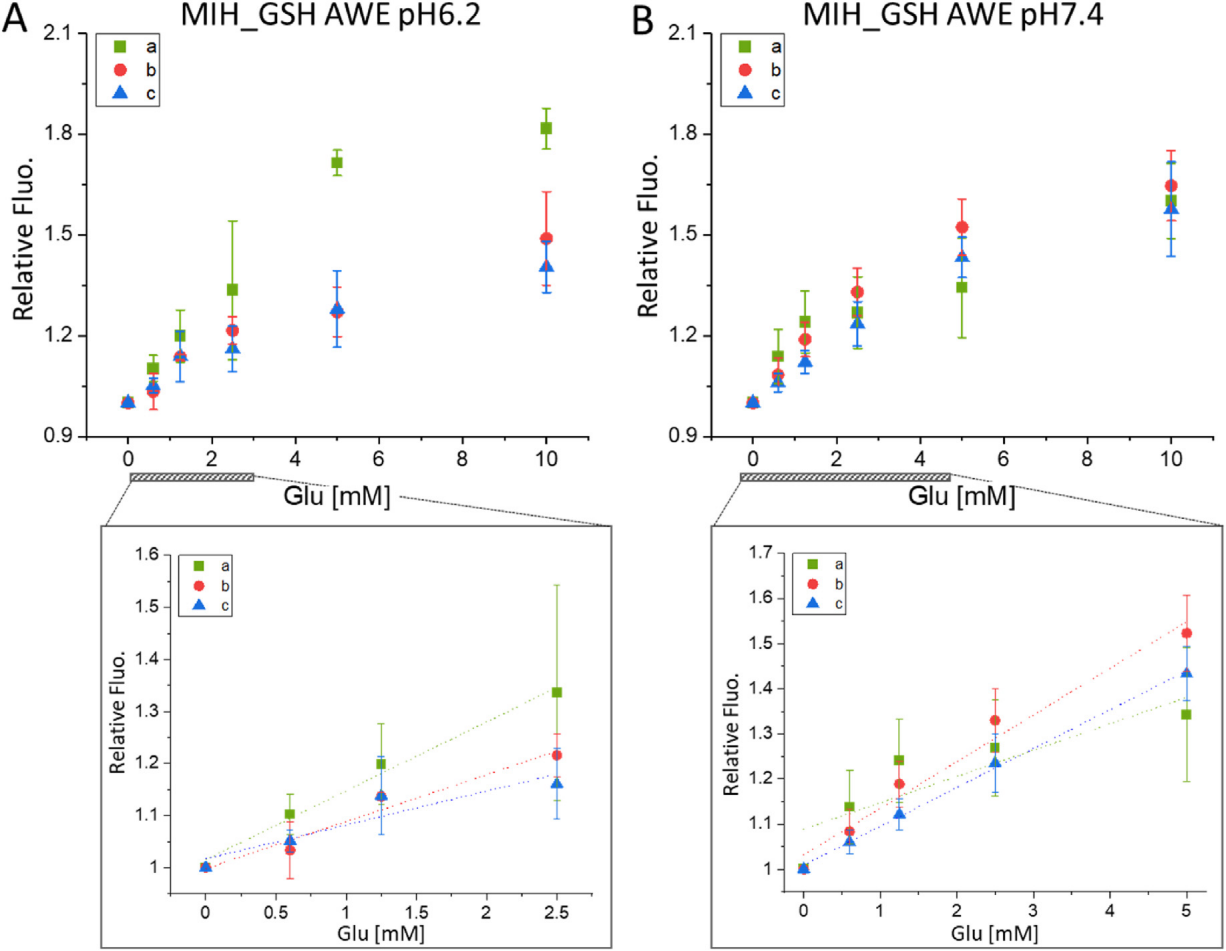
Glucose-responsiveness in AWE. Signal of MIH_GSH when treated with AWE with glucose at different concentrations (0–10 ​mM) at pH 6.2 (A) and pH 7.4 (B). The insights show the range of linearity of MIH_GSH at pH 6.2 (2.5 ​mM) and pH 7.4 (5 ​mM). MIH_GSH pH 6.2 a- R^2^ 0.97, residual sum of squares (RSS) 7.4 ​× ​10^−4^; b- R^2^ 0.96, RSS 0.001; c- R^2^ 0.87, RSS 0.002. MIH_GSH pH 7.4 a- R^2^ 0.97, RSS 0.004; b- R^2^ 0.76, RSS 0.016; c- R^2^ 0.99, RSS 2.7 ​× ​10^−4^. Values are expressed as mean ​± ​sd (n ​= ​3). Statistical analysis one-way ANOVA, significance level 0.01; with p ​< ​0.01 for both MIH_GSH pH 6.2 and pH 7.4.

**Fig. 5:**
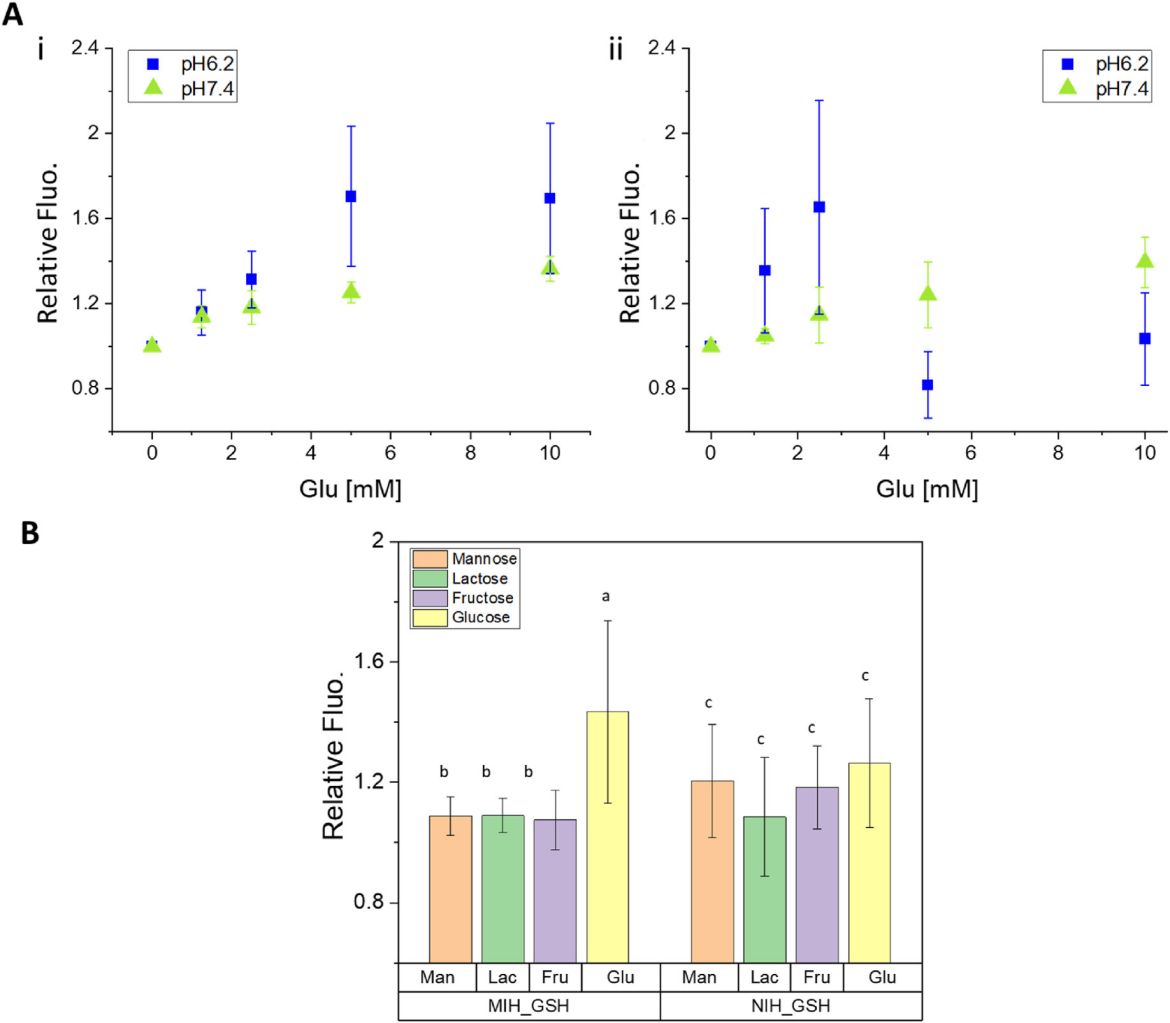
A) Glucose-responsiveness of imprinted MIH_GSH (i) and non-imprinted NIH_GSH (ii) hydrogels in the concentration range of 0–10 ​mM in presence of competitive compounds such as fructose and lactate (0.1 ​mM) mimicking the complex composition of real samples. Values are expressed as mean ​± ​sd (n ​= ​3). B) Selectivity study of MIH_GSH and NIH_GSH for glucose among other carbohydrates. The signal of the imprinted hydrogel (MIH_GSH) changes mainly in the presence of glucose (relative fluro. >1) whereas the variation of the signal when treated with the other carbohydrates is negligible (relative fluro. ca. 1). The signal of the non-imprinted hydrogel (NIH_GSH) randomly changes when treated with all carbohydrates. Statistical analysis: samples with the same superscript (a, b, c) are not significantly different among them. This result supports the improved selectivity of glucose with the imprinted hydrogel compared to the non-imprinted hydrogel (NIH_GSH). One-way ANOVA, n ​= ​9, significance level 0.05, Tukey's test). See Fig. S 10 for the complete statistical analysis. Values are expressed as mean ​± ​sd (n ​= ​9).

The variation of the material developed with the change in glucose concentration was firstly evaluated in water and PBS (Figure S 7). Results showed that with the imprinted hydrogel the fluorescent signal changes linearly with the variation of the glucose concentration in either water or PBS (R^2^ 0.99 and R^2^ 0.92, respectively). On the contrary, with non-imprinted hydrogels, the linearity of the response in water is acceptable in water (R^2^ 0.91) while it is not as good as for the imprinted hydrogel in PBS (R^2^ 0.75). The sensitivity of the hydrogels was then evaluated in a more complex environment which better represents the real situation such as AWE. The results shown in Fig. 5 refer to specimens obtained from 3 independent samples of imprinted hydrogel (a, b, and c), each tested in triplicates. The same experiment was carried out on non-imprinted hydrogels (Figure S 8). The signal measured for MIH_GSH changes with the increase of glucose maintaining good linearity at pH 7.4 up to 5 ​mM (Fig. 4-B). At pH 6.2 instead, the signal increased linearly up to 2.5 ​mM of glucose with a lower increment in the signal at higher concentrations due to the higher dissociation rate typically observed for the glucose-boronic acid reversible interaction in a neutral-acid environment (Fig. 4-A). The limited linearity observed at the concentration of glucose above 5 ​mM is ascribed to the saturation point of the synthesized hydrogels that leads to an upper limit of detection. In particular, considering the amount of AAPBA initially incorporated into the hydrogel matrix (160 ​mM each sample divided into 8 specimens ​= ​20 ​mM), the theoretical amount of glucose that can be absorbed by each specimen is between 10 ​mM and 20 ​mM depending on the formation of di- or mono-valency BA-glucose interactions. However, the adsorption of glucose could be lower than the theoretical value due to self-limitation of the interaction once close to the saturation point (10–20 ​mM). Nevertheless, considering that the concentration of glucose in chronic wounds is 1.2 ​mM and 2 ​mM in healing wounds [53], the hydrogels here presented can be used as an efficient fluorescent-based platform for glucose detection in physiological fluids such as wound exudate. In particular, the limit of detection (LOD) calculated as 3.3σ/slope, with σ as the standard deviation measured for the blank, for MIH_GSH in AWE at pH 7.4 was 0.48 ​mM of glucose and 0.54 ​mM at pH 6.2, making it suitable for the quantification of glucose in wound exudates. Statistical analysis of the results showed that at any condition (pH 6.2 and 7.4) the fluorescence signal changed significantly in response to the variation of the concentration of glucose for both MIH_GSH and NIH_GSH. Importantly, this test further confirmed the improvement achieved with the imprinting approach. Indeed the results obtained using NIH_GSH were not as promising as using MIH_GSH concerning both reproducibility (between replicates and independent specimens as shown in Figure S 8) and sensitivity (with a calculated LOD of 1.4 ​mM and 1.0 ​mM at pH 6.2 and 7.4 respectively).

Besides the improved responsiveness towards glucose concentration, lower pH influence and better reproducibility, the hypothesis behind the imprinting approach is to increase the selectivity of the detection method, favoring the formation of multivalent interaction BA-glucose using the imprinting approach. The presence of glucose during polymerization ensures the appropriate orientation of the boronic moieties in the pocket formed after template removal. This will support the multivalency interaction of glucose with BA specifically during the re-sorption experiments, limiting instead the undesired interaction with other carbohydrates as schematized in Figure S 9. For instance, mannose and lactose can only interact with one boronic acid at a time, reducing the binding affinity. Due to the similar structure, the discrimination between glucose and fructose is more complicated but the orientation of the hydroxyl groups in the latter can be exploited to restrict the influence of fructose in glucose quantification.

The glucose selectivity achieved using the imprinted MIH_GSH was evaluated and compared with the results obtained with the non-imprinted hydrogel. Firstly, the competitive binding was tested using complex solutions containing molecules usually present in physiological fluids mimicking the real scenario [53,54]. In particular, the specimens were treated with AWE solutions at pH 6.2 and 7.4 containing different concentrations of glucose in the range of 0–10 ​mM plus the constant amount (0.1 ​mM) of d-fructose and l-lactate, an α-hydroxy carboxylate metabolite present in physiological fluids that can also compete with carbohydrates interacting with BA. As shown in Fig. 5-A, the signal increased linearly up to 5 ​mM of glucose at both pHs in the case of MIH_GSH (i) while good responsiveness was observed only at pH 7.4 using the non-imprinted hydrogel (ii), indicating that imprinting strategy improves the sensitivity of the detection method for glucose.

The selectivity of the detecting approach among other carbohydrates was further evaluated. In this study, the same specimen was treated with solutions of d-mannose, d-lactose, d-fructose, and d-glucose at the same concentration (2.5 ​mM) in PBS at pH 7.4. MIH_GSH showed a remarkable selectivity for glucose compared to the NIH_GSH supporting that the imprinting procedure allows overcoming the typical low-specificity of BA-based glucose-detection approaches (Fig. 5-B). Statistic analysis confirmed the advantages achieved by employing the imprinting procedures showing that the values measured for glucose using MIH_GSH are statistically different from the values measured for d-mannose, d-lactose, and d-fructose (Figure S 10) with an imprinting factor (IF) of 1.7. The IF is a measure of the strength of interaction between the imprinted hydrogel and glucose. IF values greater than 1 indicate that the imprinting approach improved the binding efficiency between glucose and the hydrogel. IF was calculated as the ratio between the equilibrium binding capacity (Q) of the imprinted and non-imprinted hydrogels. Q was calculated using equation (1) [55]:

(1) $$Q=\frac{Ci-Cf}{M}\;V$$

where *Ci* is the initial concentration of glucose and *Cf* is the concentration of glucose after 30 ​min of treatment with the hydrogels. *M* is the mass of the specimen and *V* is the volume of the solution used for the experiment.

The apparent binding affinities of MIH_GSH and NIH_GSH for d-glucose and other carbohydrates (d-mannose, d-lactose, d-fructose) were determined by measuring the variation of the fluorescent signal of the hydrogels when treated with different concentrations of the tested analytes (0–10 ​mM, PBS pH 7.4). As shown in Figure S 11, the fluorescence decreased only when treated with glucose and fructose, whereas an increase of the signal was observed with mannose and lactose confirming the improved selectivity of the proposed imprinted hydrogel. The association binding constant (Ka) was calculated from Lineweaver-Burk plots of glucose and fructose, for which the signal decreased linearly with the increase of analyte concentration (Figure S 12) [56]. The Ka between MIH_GSH and glucose was found to be 82.9 ​M^-1^, more than 6-fold higher than the Ka calculated for NIH_GSH (13.1 ​M^-1^) confirming the improved affinity of glucose for the recognition boronic acid moieties when the latter is specifically oriented thank to the imprinting procedure. The high Ka calculated for NIH_GSH-fructose (416.9 ​M^-1^) confirmed the high affinity between boronic acid moieties and fructose rather than glucose as reported in the literature [57]. On the contrary, the change in fluorescence intensity was too small for an accurate determination of Ka between MIH_GSH and fructose suggesting a limited affinity of the imprinted hydrogel for carbohydraes diffrent than glucose.

### 3.4 Glucose-sensing on wound exudates

The sensitivity of the imprinted hydrogel MIH_GSH was further tested using wound exudate samples collected from hospitalized patients. Depending on the progression of the wound healing process, the exudate can have different properties such as color, clarity, viscosity, etc. In particular, the presence of blood in the wound exudate is a normal sign of the inflammatory phase but if it lasts for several days, it indicates an impairment of the healing process (stress, further trauma, infections, or underlying pathologies). The presence of blood and biological material in the wound exudate could be a limitation for well-known colorimetric assays due to the obvious influence on the background signal, which affects the sensitivity and robustness of the quantification. In this experiment, we used sanguineous wound exudate (5 independent samples) aiming to evaluate the efficiency in glucose detection of our proposed method in the worst condition (Figure S 13). The concentrations determined with MIH_GSH were compared with the colorimetric enzymatic-based assay widely used for the quantification of glucose. For these tests, 6 specimens of MIH_GSH were treated with 100 ​μL of wound exudate (WE) and after 30 ​min the signal was measured, and the amount of glucose present in the samples was calculated using the calibration curve of glucose in AWE at pH 7.4. The pH 7.4 was chosen since it corresponds to the pH of the real WE samples. The concentration of glucose (mM) quantified for each WE sample is reported in Table 1. The average concentration of glucose determined with MIH_GSH for the WE samples shows no significant difference to the one obtained with the colorimetric method, indicating a good reproducibility and robustness of the detection approaches. However, in the case of the colorimetric assay, the WE had to be diluted 10 times to have a readable signal since, if not diluted, the values measured were above the threshold of the instrument (>10 a.u. for the bloody sample) as well as above the reliable measurement (>1 a.u.) as reported in the Material and Methods. On the other hand, no sample pre-treatment was required for the glucose quantification using MIH_GSH highlighting the advantage achieved using fluorescent-based approaches in detecting the targeted analyte in biological samples [58]. Human blood is characterized by peculiar UV-spectrum due to the presence of proteins (e.g. hemoglobin, albumin) and cells absorption peaks in the UV (200–400 ​nm) and UV–Vis (400–700 ​nm) regions but that do not have fluorescent properties. Hence, the analyte should be isolated for biological samples (by centrifugation or alternative techniques [59]) to be trustfully quantified by colorimetric assay whereas it is not required when using fluorescent-based approaches [31]. Moreover, the colorimetric assay used as a benchmark in this study is enzymatic-based. This implies that the mixture of reagents has to be reconstituted just before the experiment and it is stable for only a couple of weeks if stored at 4 ​°C and for a few months if frozen. On the contrary, due to its proven stability, MIH_GSH can be reused for several tests without particular precautions but simply keep the hydrogel wet. Due to its simple operation procedure, cost-efficiency, sensitivity, selectivity the here presented fluorescent-based detection mechanism can be used as it is to quantify glucose directly from complex biological samples (i.e. bloody wound exudate). However, MIH_GSH can even be applied for the fabrication of more sophisticated devices. For example, we are currently investigating the possibility of incorporating it into optical fibers designing a wearable device capable of monitoring the glucose level in chronic wounds to constantly monitor the healing process.

## 4 Conclusion

In this work, we proposed a cost-efficient fluorescent-based hydrogel for the detection of glucose with high sensitivity. The method is based on BA moieties which are covalently incorporated into the hydrogel network. The change in stiffness of the hydrogel upon glucose adsorption is measured as fluorescence change of the incorporated Tht. The stability of the hydrogel was confirmed over time and the pH-dependency, commonly observed for BA-based approaches, was limited thanks to the incorporation of DMAEMA into the hydrogel network. The imprinting approach favoring the appropriate orientation of BA moieties into the glucose-designed pockets improved the selectivity of the detection in respect to other carbohydrates such as fructose, mannose, and lactose. A binding affinity of 82.9 ​M^-1^ was determined between glucose-MIH_GSH, which was 6-fold higher than the Ka determined for the non-imprinted hydrogel with a calculated imprinting factor of 1.7. The imprinting strategy proposed proved to be a useful tool to overcome one of the main drawbacks of boronic acid-based detection approaches, which is the low selectivity for glucose. Moreover, the proposed fluorescent-based approach not only allowed to achieve a good sensitivity (LOD of 0.54 and 0.48 ​mM in AWE at pH 6.2 and 7.4 respectively), reliability and reproducibility, but it is an affordable and user-friendly method that does not require expensive chemicals and complicated technologies. The fluorescent-based approach ensures the direct analysis of concentrated, colored, blurry samples without sample manipulation which is required if using colorimetric assays. Given the above-mentioned advantages, the presented fluorescent-based imprinted hydrogel for the detection of glucose could pave the way for the development of cost-efficient glucose quantification in complex human samples, such as bloody wound exudate.

## Funding

This work was supported by grant #2018-532 of the Strategic Focal Area “Personalized Health and Related Technologies (PHRT)” of the 10.13039/501100003006ETH Domain.

## Data availability

The processed data required to reproduce these findings are available to download from 10.5281/zenodo.6006709.

## Credit author statement

Giorgia Giovannini: Conceptualization; Data curation; Formal analysis; Investigation; Writing – original draft; Writing – review & editing. Paolo Cinelli: Samples collection; Writing-review; Luciano F. Boesel: Data curation; Writing – review & editing; Supervision; Funding acquisition; Project administration; René M. Rossi: Writing – review & editing; Supervision; Funding acquisition; Project administration.

## Declaration of competing interest

The authors declare that they have no known competing financial interests or personal relationships that could have appeared to influence the work reported in this paper.
