# Supplementary material for: Thioflavin-modified molecularly imprinted hydrogel for fluorescent-based non-enzymatic glucose detection in wound exudate
Source: Mater Today Bio. 2022 Apr 9;14:100258. doi: 10.1016/j.mtbio.2022.100258 (PMC9034389; doi:10.1016/j.mtbio.2022.100258)
Supplement: Multimedia component 1 [file mmc1.docx]

**Supporting information**

Thioflavin-Modified Molecularly Imprinted Hydrogel for Fluorescent-Based Non-Enzymatic Glucose Detection in Wound Exudate

Giorgia Giovannini^a*^, Paolo Cinelli^b^, Luciano F. Boesel^a^ and René M. Rossi^a*^

a Empa, Swiss Federal Laboratories for Materials Science and Technology, Laboratory for Biomimetic Membranes and Textiles, Lerchenfeldstrasse 5, CH-9014, St.Gallen, Switzerland

^b^ Department of Trauma, University of Zurich, Zurich, Switzerland.

^*^ Corresponding authors

[1 Hydrogel formulation 2](#_Toc92532023)

[2 Hydrogel characterization 2](#_Toc92532024)

[FT-IR analysis 2](#_Toc92532025)

[*UV-Vis and fluorescent analysis* 2](#_Toc92532026)

[Swelling analysis 3](#_Toc92532027)

[Definition of the detection time 3](#_Toc92532028)

[pH-stability 4](#_Toc92532029)

[Fluorescent signal stability between consecutive experiments 4](#_Toc92532030)

[3 Evaluation of glucose-responsiveness 5](#_Toc92532031)

[Glucose detection in water and PBS 5](#_Toc92532032)

[Glucose responsiveness of NIH_GSH in AWE at pH 6.2 and 7.4 5](#_Toc92532033)

[4 Evaluation of glucose-selectivity 6](#_Toc92532034)

[5 Wound exudates 7](#_Toc92532035)

# Hydrogel formulation

Table S 1: Final concentration of each component in the chosen formulation.

|  | AA  (% wt) | Bis  (% wt) | DMAEMA  (mM) | Tht  (µM) | AAPBA  (mM) | AAPBA-Glu  (mM) | TEMED  (mM) | APS  (mM) |
| --- | --- | --- | --- | --- | --- | --- | --- | --- |
| MIH_GSH | 5.7 | 0.3 | 4.5 | 250 | / | 160 | 0.7 | 2.5 |
| NIH_GSH | 5.7 | 0.3 | 4.5 | 250 | 160 | / | 0.7 | 2.5 |

Table S 2: Leakage test confirming the stability of Tht once entrapped in the hydrogel matrix. The amount of Tht that leaked out the hydrogel at each time point (1, 2, 3, 24, and 48 hours) was quantified by measuring the absorption signal of Tht in the water collected at each washing step (1-5). The amount of Tht (nmol) was calculated using the calibration curve of Tht in water (slope 0.0073; intercept 0.0042; R^2^ 0.99). Given that 500 nmol (250 µM in 2 mL) were added for the hydrogel formation, we calculated the % of Tht that leaked out of the hydrogels during washing. As observed, after 4h (washing 3) no Tht was detected in the water in the following steps (washing 4 and 5). Moreover, considering that only 3.72% (18.6 nmol) and 5.58% (5.58 nmol) of Tht leaked out respectively MIG_GSH and NIH_GSH during the entire process, we concluded that Tht is stably incorporated inside the hydrogel matrix even if only physically entrapped.

|  | Washing1 (1h) | Washing2 (2h) | Washing3 (4h) | Washing4 (24h) | Washing5 (48h) |
| --- | --- | --- | --- | --- | --- |
| Tht that leaked out the of hydrogel (nmol) | | | | | |
| MIH_GSH | 15.0 | 3.00 | 1.00 | -1.00 | -1.54 |
| NIH_GSH | 19.0 | 8.00 | 0.00 | -0.98 | -1.43 |
| Tht that leaked out of the hydrogel (%) | | | | | |
| MIH_GSH | 2.92 | 0.61 | 0.19 |  |  |
| NIH_GSH | 3.88 | 1.61 | 0.08 |  |  |
| Total amount of Tht that leaked out of the hydrogel | | | | | |
| MIH_GSH | 18.6 nmol | 3.72 % |  |  |  |
| NIH_GSH | 27.9 nmol | 5.58 % |  |  |  |

# Hydrogel characterization

## FT-IR analysis


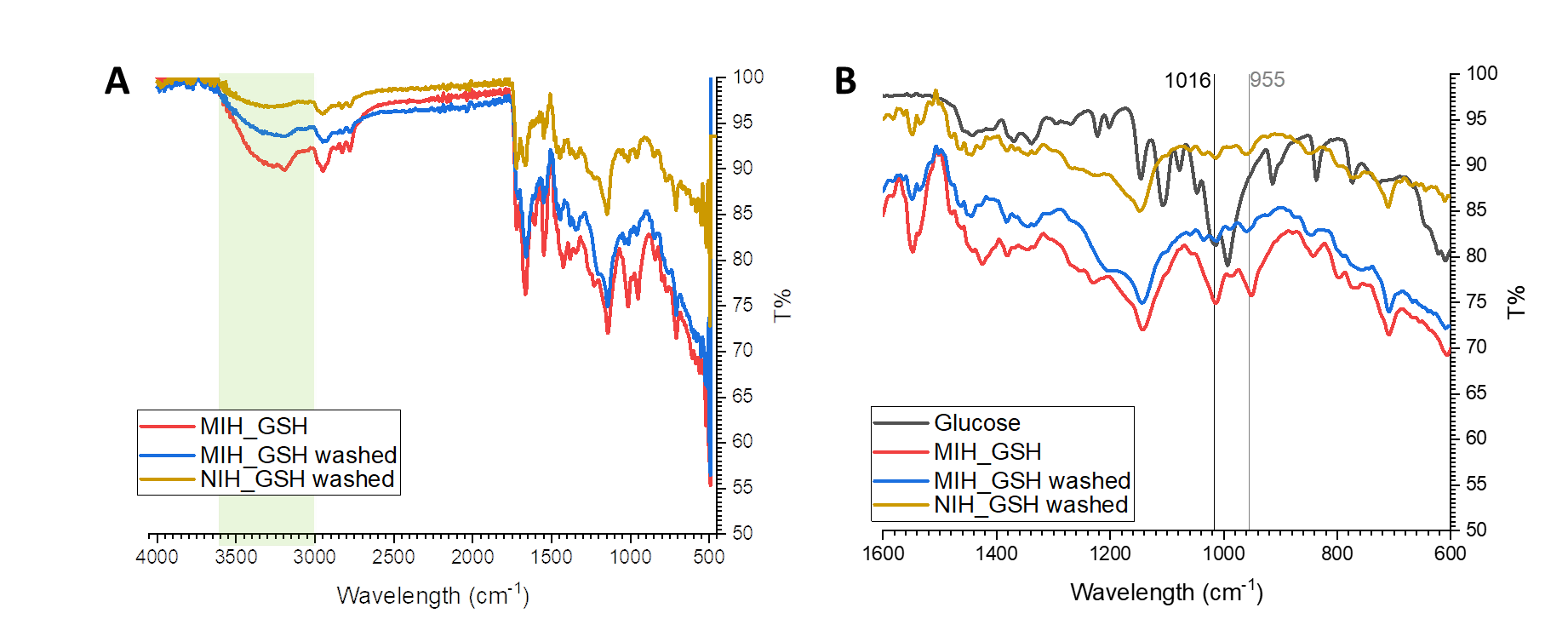


Figure S 1: FT-IR analysis of the glucose-sensitive hydrogels. The broad peak of the OH starching in the 3600-3400 cm-1 region indicates the presence of intermolecular H-bonding in the hydrogel structure (A). The disappearance of peaks at 1016 and 955 cm-1 proved the successful removal of glucose from the imprinted hydrogel after washing (B).

## UV-Vis and fluorescent analysis


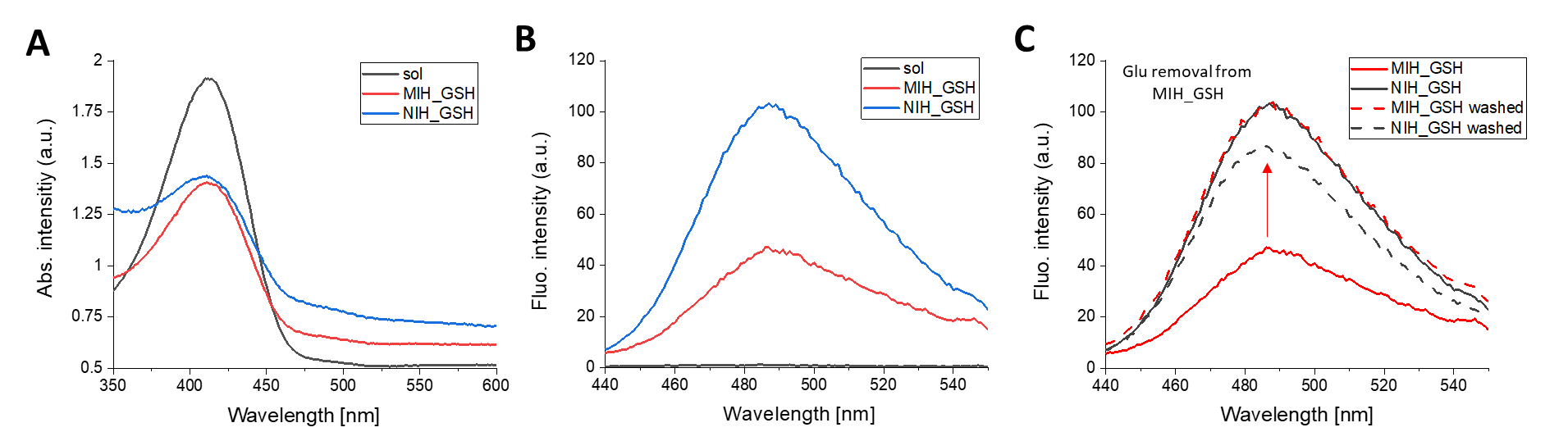


Figure S 2: UV-Vis analysis of Tht T in solution and embedded in the hydrogel matrix (A). Fluorescent scan of Tht T in solution and embedded in hydrogel when exited at 410 nm (B). An increase in the fluorescent peak at 480 nm was measured for MIH_GSH after the removal of glucose proving the stiffening effect of the H-bonding between boronic acid moieties (C).

## Swelling analysis


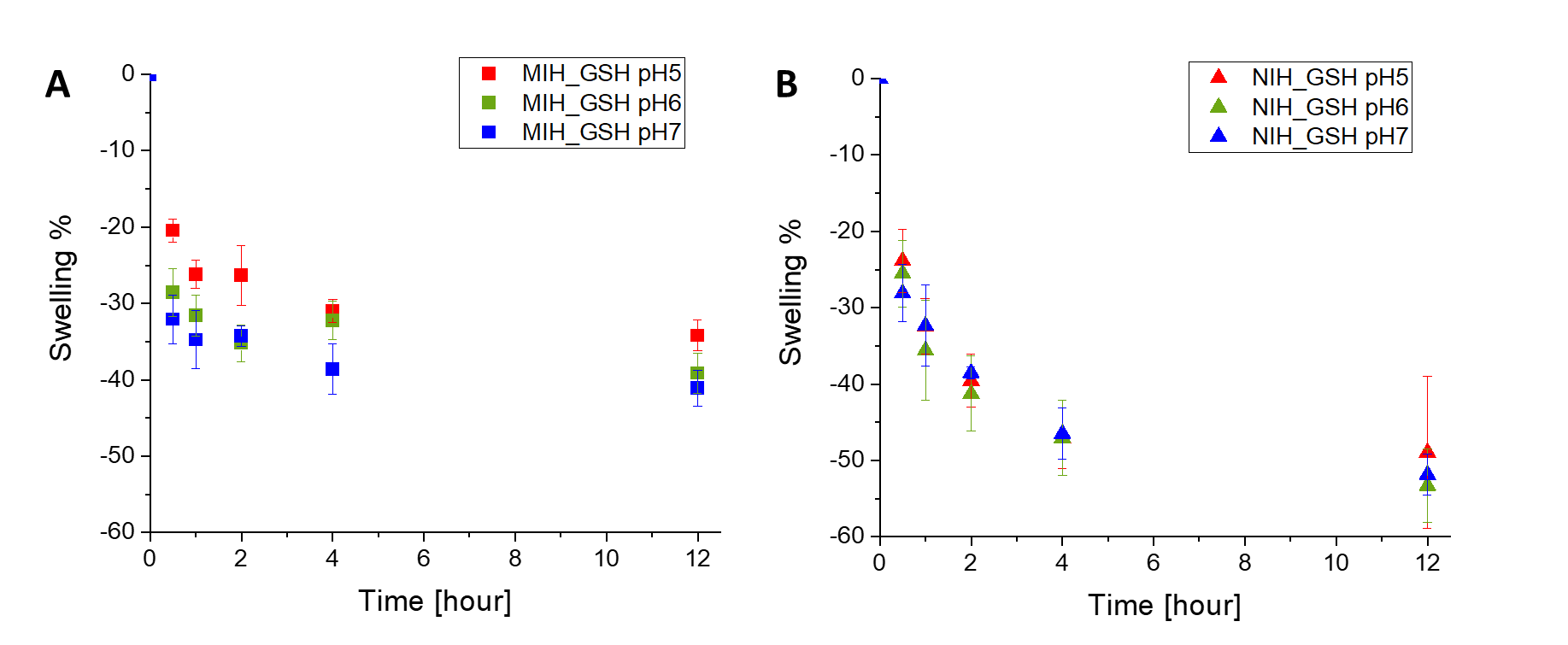


Figure S 3: Swelling of imprinted (MIH_GSH) and non-imprinted (NIH_GSH) hydrogels in PBS at pH5, 6, and 7 showing the negligible influence of the pH to the sweölling of the hydrogel achieved using the cationic monomers (DMAEMA) in the hydrogel formulation. The %swelling was calculated considering the variation of the weight at each time point to the initial weight. Values are reported as mean ± sd (n=3).

## Definition of the detection time


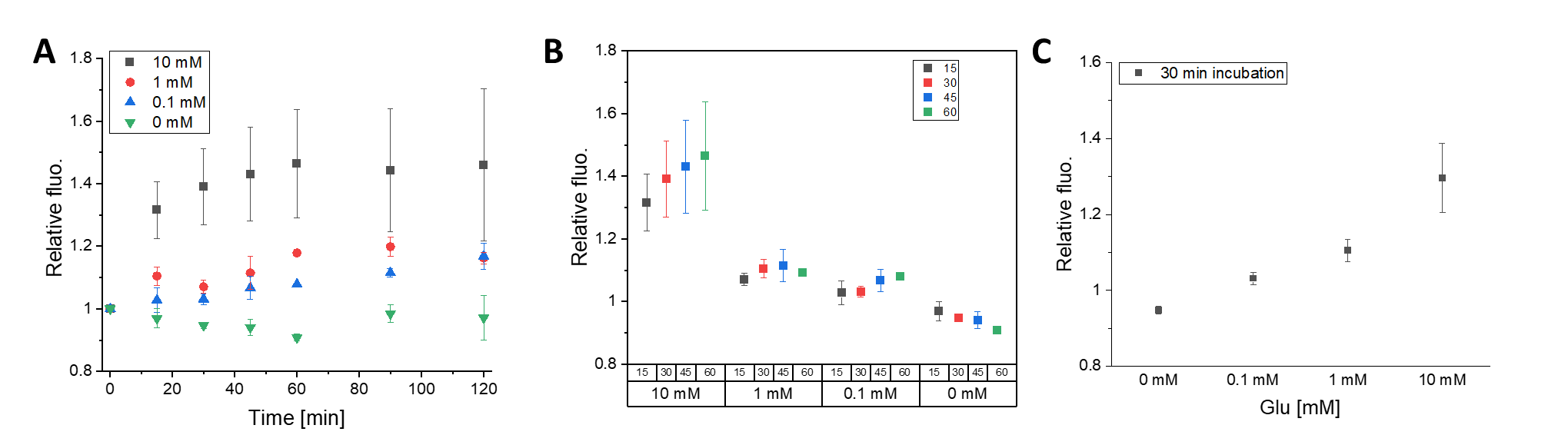


Figure S 4: Time-related measurements indicating the time required to stabilize the signal for MIH_GSH (A). Statistical analysis of the signal measured indicating that the signals for each glucose concentration at different time points (15, 30, 45, and 60 minutes) are not significantly different ((One-way ANOVA, n=2, significance level 0.05, Tukey's test, Levene's test, actual power, B). Linear response of signal variation after 30 minutes of equilibration at different glucose concentrations (R^2^=0.9, C). Values are reported as mean ± sd (n=2).

## pH-stability


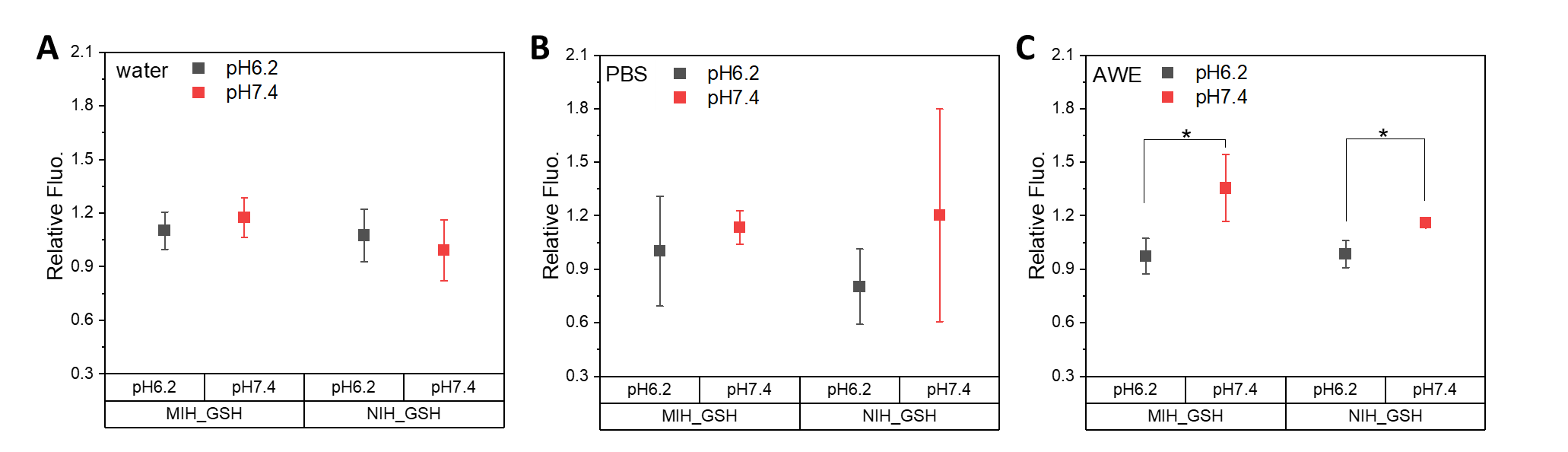


Figure S 5: Evaluation of glucose (5 mM) detection in a different environment (water - A, PBS - B, and AWE - C) at different pHs (i.e. 6.2 and 7.4). Two-sample t-test (level of significance 0.05) indicated that there is no significant difference in the relative fluorescence values measured for MIH-GSH and NIH_GSH respectively, in water and PBS, at pH6.2 and 7.4. On the contrary, a statistical difference was found for MIH_GSH and NIH_GSH when tested in AWE at pH 6.2 and 7.4 (C, * p<0.05 MIH_GSH p = 0.007 and NIH_GSH p = 0.002). Values are reported as mean ± sd (n=3).

## Fluorescent signal stability between consecutive experiments

Figure S 6: stability of the signal before and after the glucose treatment. The signal measured before (start) and after (end) the experiment for the imprinted and non-imprinted hydrogel in AWE at pH 6.2 and 7.4 was found not to be statistically different (Two-sample t-test, n=3, significance level 0.05). Values are reported as mean ± sd (n=3).

# Evaluation of glucose-responsiveness

## Glucose detection in water and PBS


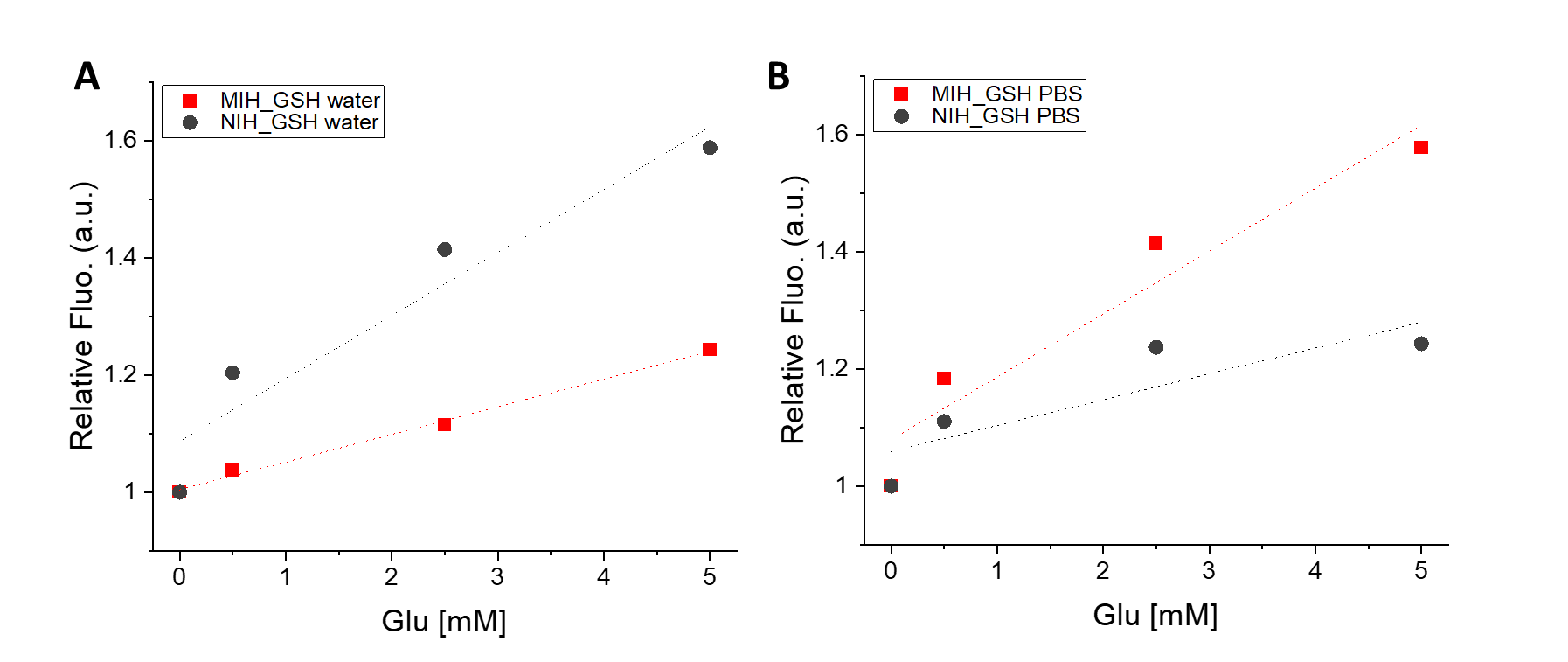


Figure S 7: Evaluation of the response of imprinted and non-imprinted hydrogels when treated with water (A) and PBS (B) solution of glucose at different concentraitons (0, 0.5, 2.5 and 5 mM). MIH_GSH in water R^2^ 0.99, residual sum of squares (RSS) 0.00016; NIH_GSH in water R^2^ 0.92, RSS 0.016; MIH_GSH in PBS R^2^ 0.92, RSS 0.015; NIH_GSH in PBS R^2^ 0.75, RSS 0.010.

## Glucose responsiveness of NIH_GSH in AWE at pH 6.2 and 7.4


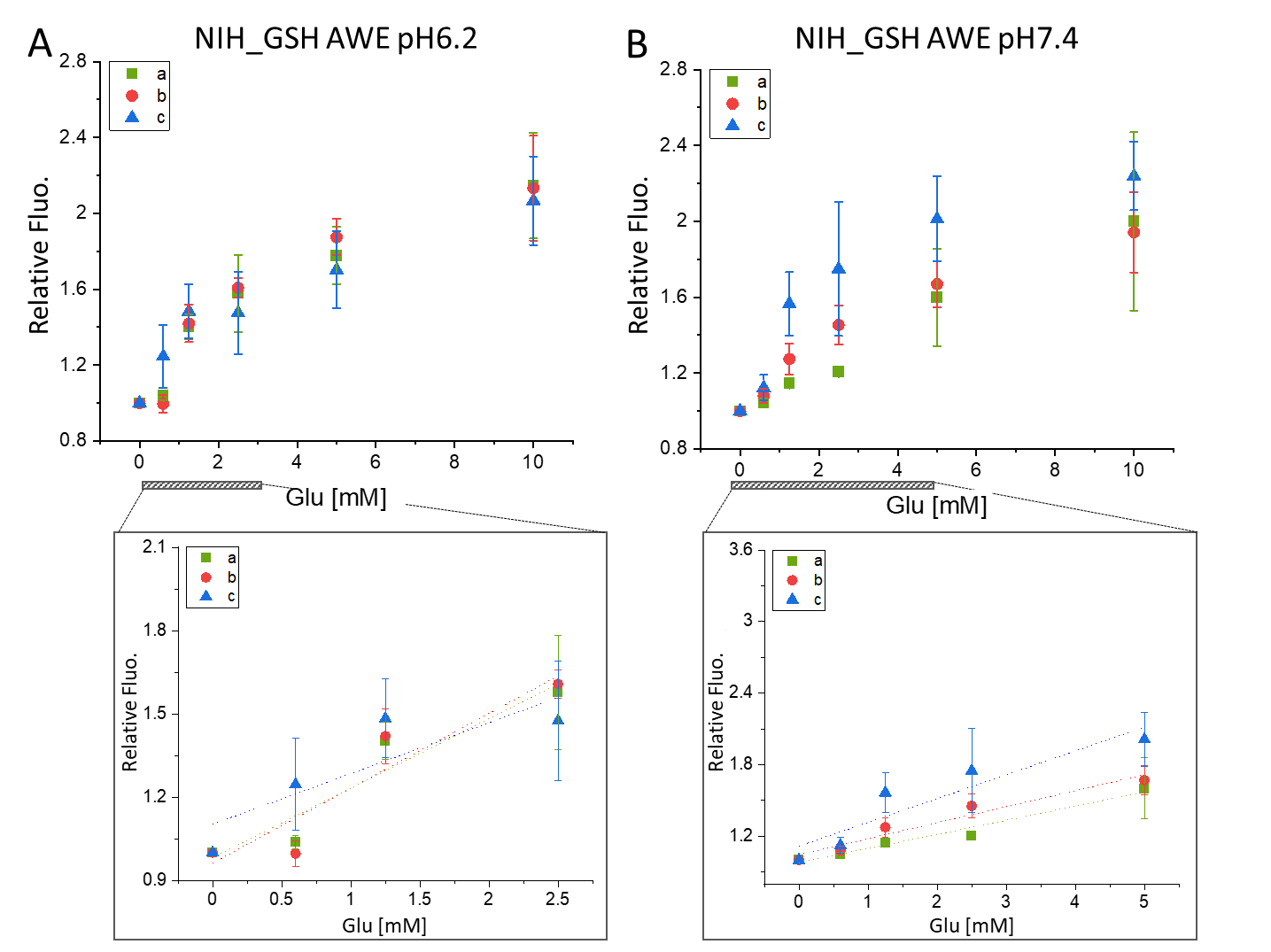


Figure S 8: Glucose-responsiveness in AWE. Singal of NIH_GSH when treated with AWE with Glu at different concentrations (0-10 mM) at pH 6.2 (A) and pH 7.4 (B). The insights show that the linearity is partially preserved at pH7.4 while it is completely lost at pH6.2. NIH_GSH pH6.2 a- R^2^ 0.91, RSS 0.022; b- R^2^ 0.88, RSS 0.033; c- R^2^ 0.73, RSS 0.042. NIH_GSH pH7.4 a- R^2^ 0.97, RSS 0.006 ; b- R^2^ 0.95, RSS 0.015; c- R^2^ 0.87, RSS 0.094. Values are reported as mean ± sd (n=3) of three samples (a,b, and c). Statistical analysis one-way ANOVA, significance level 0.01; with p<0.01 for both NIH_GSH pH 6.2 and pH 7.4.

# Evaluation of glucose-selectivity


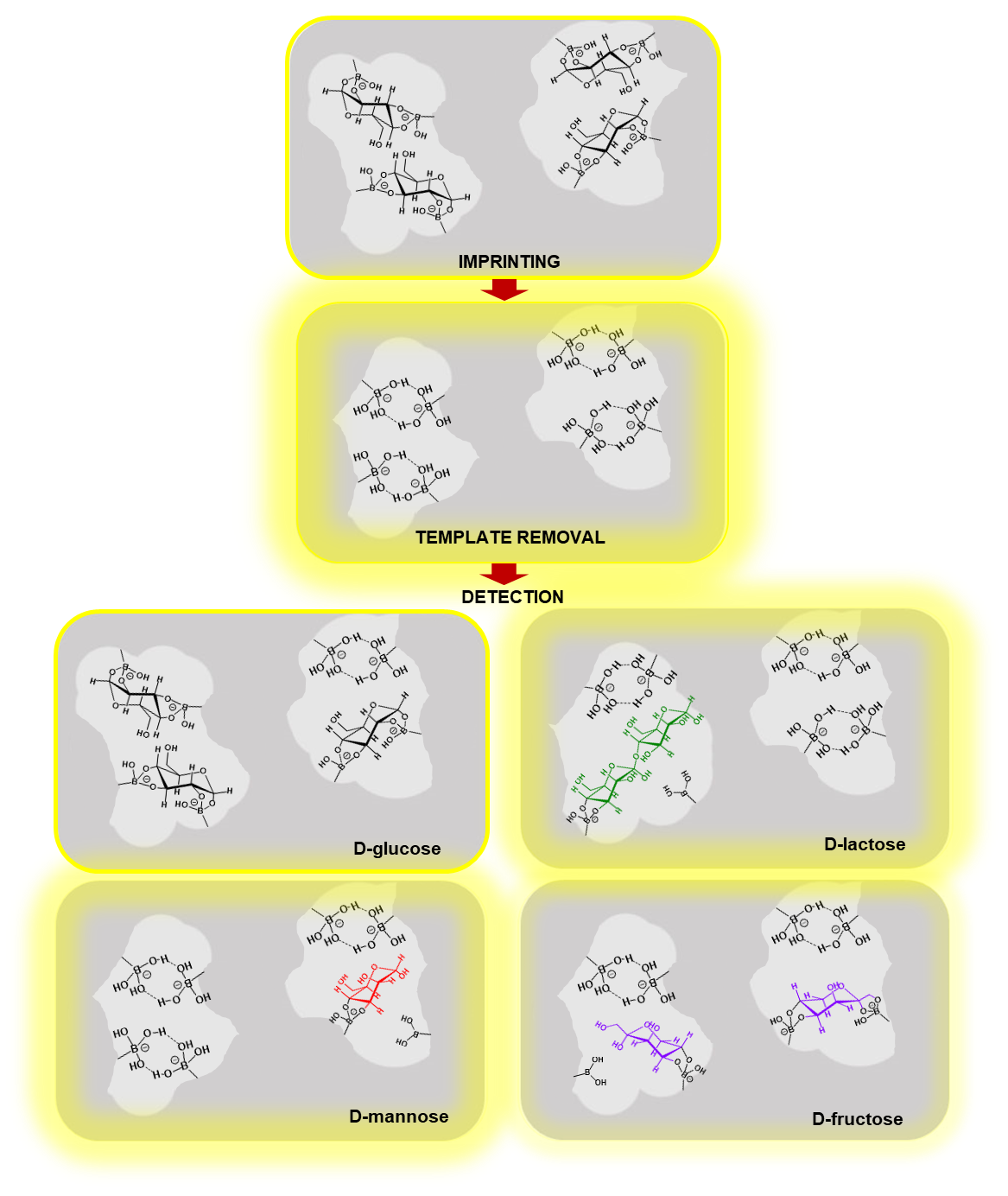


Figure S 9: Scheme of the advantages obtained in terms of selectivity for glucose detection among other carbohydrates exploiting the imprinting procedure.


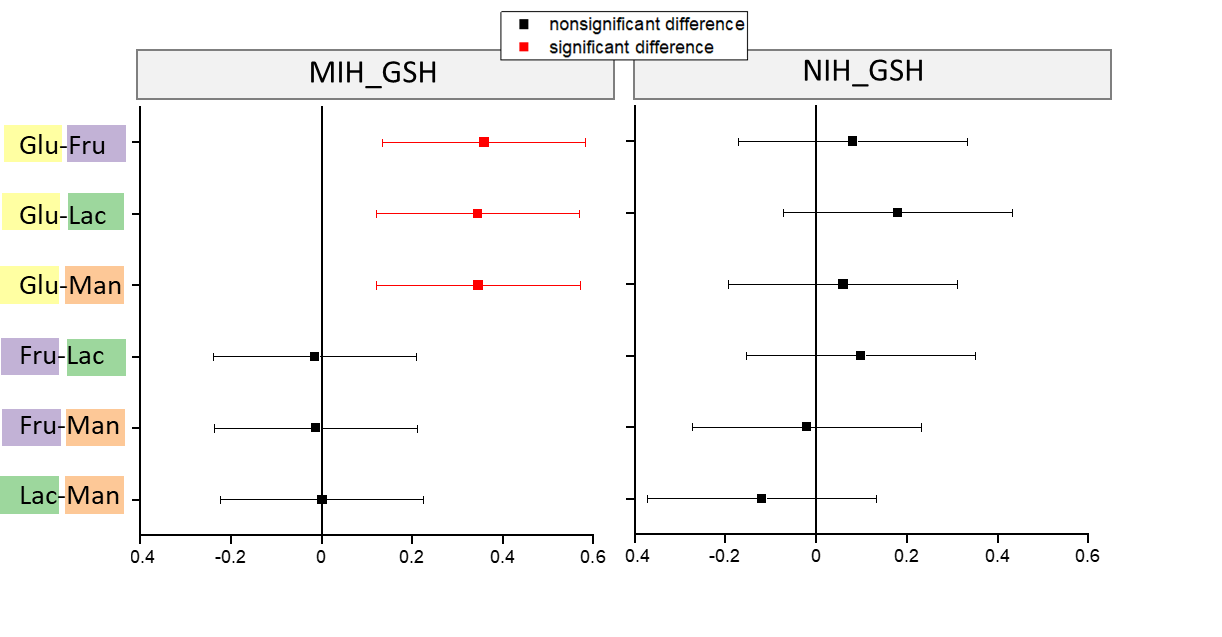


Figure S 10: Statistical analysis of the results: the signal measured for Glu is statistically different to the one measured for the other carbohydrate when using MIH_GSH supporting the improved selectivity of glucose with the imprinted hydrogel compared to the non-imprinted hydrogel for which the signals measured after treatment with the different molecules are not statistically different. One-way ANOVA, n=3, significance level 0.05, Tukey's test, Levene's test, actual power).


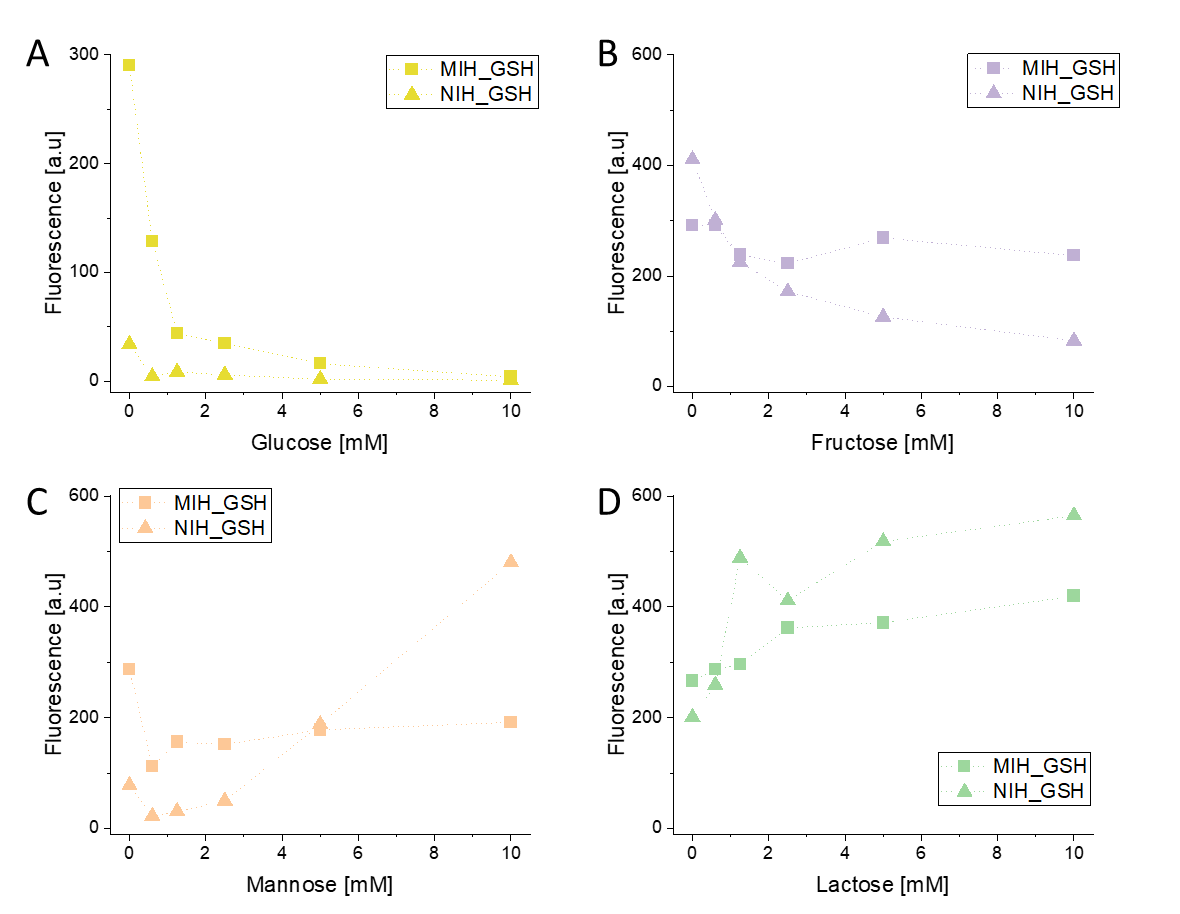


Figure S 11: Variation of the fluorescent signal of MIH_GSH (square) and NIH_GSH (triangle) when treated with different carbohydrates (A-glucose; B-fructose; C-lactose; D-mannose) in the concentration range of 0, 0.6, 1.25, 2.5, 5, and 10 mM (in PBS pH 7.4). The fluorescent signal decreases only when treated with glucose and fructose (A and B) highlighting the improved selectivity achieved with the proposed hydrogels.


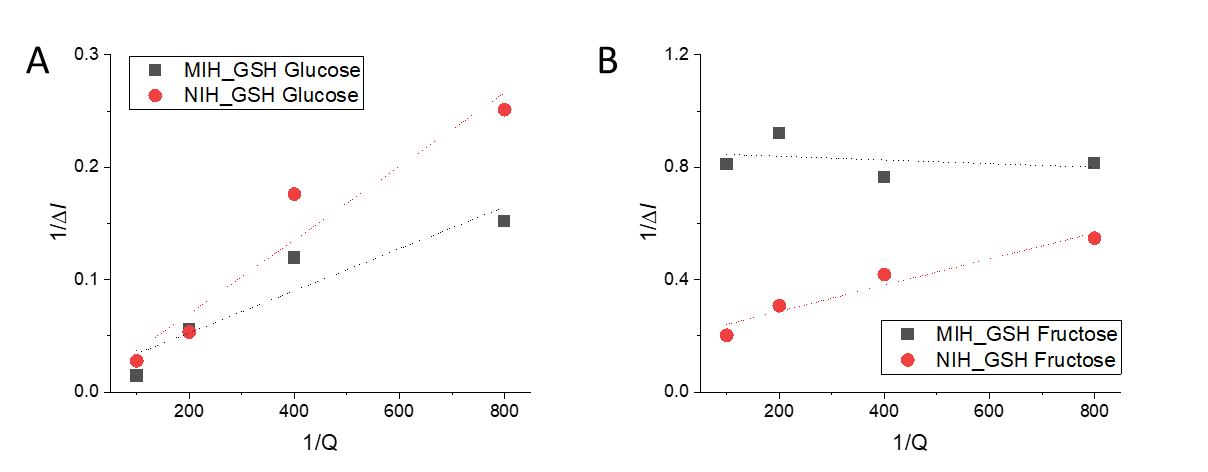


Figure S 12: Lineweaver-Burk plots reporting 1/[ΔI] against 1/[Q], where ΔI is the variation of the fluorescent signal and Q is the concentration (in M) of glucose (A) or fructose (B). LB plots are suitable to evaluate the binding affinity starting from fluorescent signals. The plots are used to estimate Ka (Ka = intercept/slope). A) MIH_GSH: slope 1.87 E-4; intercept 1.55 E-2; R^2^ 0.87. NIH_GSH: slope 3.28 E-4; intercept 4.29 E-3; R^2^ 0.93. B) MIH-GSH: slope -6.49 E-5; intercept 0.825; R^2^ 0.09. NIH-GSH: slope 4.66 E-4; intercept 0.19; R^2^ 0.94

# Wound exudates

Wound exudates were collected from negative-pressure wound therapy sponges upon ex-change in the operation room. The wound exudate was collected by squeezing vacuum-assisted closure (VAC) sponges previously applied on unhealed wounds of different patients. Depending on the progression of the wound healing process, the exudate can have different properties such as color, clarity, viscosity, etc. In particular, the presence of blood in the wound exudate is a normal sign of the inflammatory phase but if it lasts for several days, it indicates an impairment of the healing process (stress, further trauma, infections, or underlying pathologies).


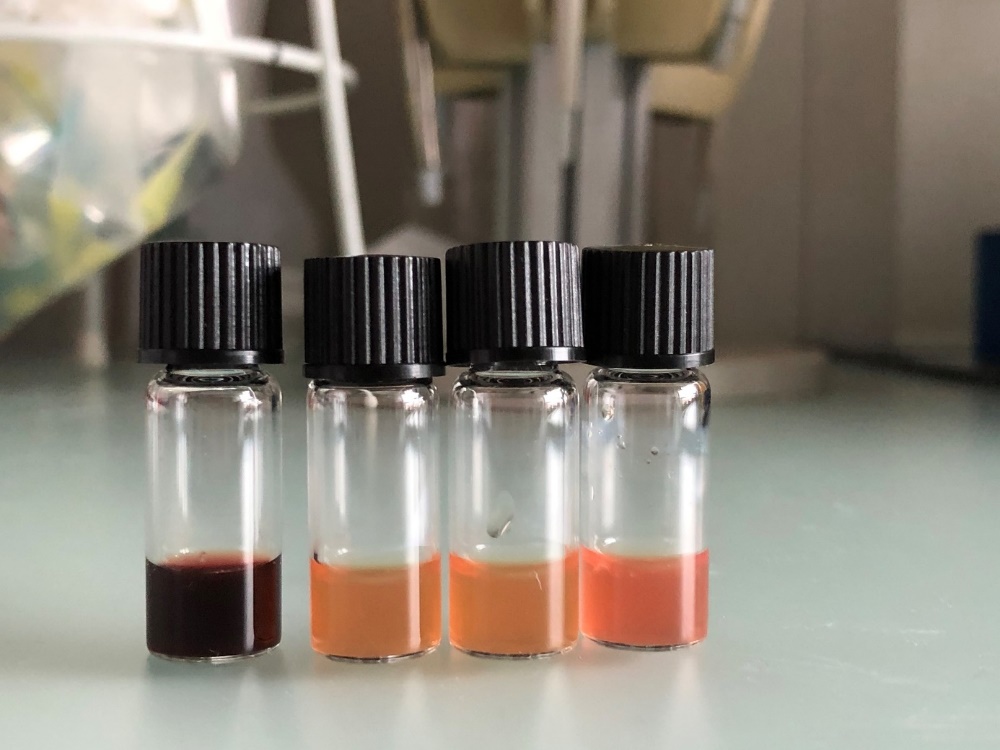


Figure S 13: Wound exudate samples.
